# Temporal Dynamics of CD8^+^ T Cell Effector Responses during Primary HIV Infection

**DOI:** 10.1371/journal.ppat.1005805

**Published:** 2016-08-03

**Authors:** Korey R. Demers, George Makedonas, Marcus Buggert, Michael A. Eller, Sarah J. Ratcliffe, Nilu Goonetilleke, Chris K. Li, Leigh Anne Eller, Kathleen Rono, Lucas Maganga, Sorachai Nitayaphan, Hannah Kibuuka, Jean-Pierre Routy, Mark K. Slifka, Barton F. Haynes, Andrew J. McMichael, Nicole F. Bernard, Merlin L. Robb, Michael R. Betts

**Affiliations:** 1 Department of Microbiology, Perelman School of Medicine, University of Pennsylvania, Philadelphia, Pennsylvania, United States of America; 2 Center for Infectious Medicine, Department of Medicine, Karolinska Institute, Karolinksa University Hospital Huddinge, Stockholm, Sweden; 3 U.S. Military HIV Research Program, Walter Reed Army Institute of Research, Silver Spring, Maryland, United States of America; 4 Henry M. Jackson Foundation for the Advancement of Military Medicine, Bethesda, Maryland, United States of America; 5 Department of Biostatistics and Epidemiology, Perelman School of Medicine, University of Pennsylvania, Philadelphia, Pennsylvania, United States of America; 6 Medical Research Council Human Immunology Unit, Weatherall Institute of Molecular Medicine, University of Oxford, Oxford, England; 7 Walter Reed Project-Kenya, Kenya Medical Research Institute, Kericho, Kenya; 8 Mbeya Medical Research Centre, Mbeya, Tanzania; 9 Department of Retrovirology, United States Army Medical Component, Armed Forces Research Institute of Medical Sciences (USAMC-AFRIMS), Bangkok, Thailand; 10 Makerere University Walter Reed Project, Makerere University Medical School, Kampala, Uganda; 11 Division of Hematology & Chronic Viral Illness Service, McGill University Health Centre, Montréal, Québec, Canada; 12 Division of Neuroscience, Oregon National Primate Research Center, Oregon Health and Science University, Beaverton, Oregon, United States of America; 13 Duke Human Vaccine Institute, Duke University, Durham, North Carolina, United States of America; 14 NDM Research Building, Old Road Campus, University of Oxford, Oxford, United Kingdom; 15 Research Institute of the McGill University Health Centre, Montréal, Québec, Canada; Vaccine Research Center, UNITED STATES

## Abstract

The loss of HIV-specific CD8^+^ T cell cytolytic function is a primary factor underlying progressive HIV infection, but whether HIV-specific CD8^+^ T cells initially possess cytolytic effector capacity, and when and why this may be lost during infection, is unclear. Here, we assessed CD8^+^ T cell functional evolution from primary to chronic HIV infection. We observed a profound expansion of perforin^+^ CD8^+^ T cells immediately following HIV infection that quickly waned after acute viremia resolution. Selective expression of the effector-associated transcription factors T-bet and eomesodermin in cytokine-producing HIV-specific CD8^+^ T cells differentiated HIV-specific from bulk memory CD8^+^ T cell effector expansion. As infection progressed expression of perforin was maintained in HIV-specific CD8^+^ T cells with high levels of T-bet, but not necessarily in the population of T-bet^Lo^ HIV-specific CD8^+^ T cells that expand as infection progresses. Together, these data demonstrate that while HIV-specific CD8^+^ T cells in acute HIV infection initially possess cytolytic potential, progressive transcriptional dysregulation leads to the reduced CD8^+^ T cell perforin expression characteristic of chronic HIV infection.

## Author Summary

Previous studies have demonstrated that HIV-specific CD8^+^ T cells are critical for the initial control of HIV infection. However, this control is typically incomplete, being able to neither clear infection nor maintain plasma viremia below undetectable levels. Mounting evidence has implicated CD8^+^ T cell cytotoxic capacity as a critical component of the HIV-specific response associated with spontaneous long-term control of HIV replication. CD8^+^ T cell cytotoxic responses are largely absent in the vast majority of HIV chronically infected individuals and it is unclear when or why this functionality is lost. In this study we show that HIV-specific CD8^+^ T cells readily express the cytolytic protein perforin during the acute phase of chronic progressive HIV infection but rapidly lose the ability to upregulate this molecule following resolution of peak viremia. Maintenance of perforin expression by HIV-specific CD8^+^ T cells appears to be associated with the expression level of the transcription factor T-bet, but not with the T-bet paralogue, Eomes. These findings further delineate qualitative attributes of CD8^+^ T cell-mediated immunity that may serve as targets for future HIV vaccine and therapeutic research.

## Introduction

CD8^+^ T cells play a central role in the control of HIV replication. During acute infection the emergence of HIV-specific CD8^+^ T cells correlates with resolution of peak viremia [1, 2], and in the nonhuman primate model experimental depletion of CD8^+^ T cells prior to infection with simian immunodeficiency virus delays resolution of acute viremia until the CD8^+^ T cell pool is reconstituted [3]. Further evidence of the immunologic pressure exerted by CD8^+^ T cells is manifest by CTL escape mutations throughout all phases of HIV infection and the association of certain MHC class I alleles with superior control of viral replication [4–9]. However, for the vast majority of infected individuals control is incomplete and ultimately fails in the absence of therapy. A better understanding of the CD8^+^ T cell response to HIV may inform the design of vaccines, therapeutics, or eradication strategies designed to stimulate or potentiate the natural response to infection resulting in better, if not complete, control.

The CD8^+^ T cell response to viral infection is multifaceted, including the ability to proliferate, produce multiple cytokines and chemokines, degranulate, and induce cytolysis upon contact with infected targets [10]. During chronic progressive infection, HIV-specific CD8^+^ T cells have impaired proliferative potential [11–13], are less capable of multifunctional responses [14, 15], and have reduced cytotoxic capacity [16–20]. The primary mechanism by which CD8^+^ T cells kill virally infected cells is via exocytosis of granules containing the cytolytic proteins perforin and granzyme B [21, 22]. Control of HIV viremia has been associated with the ability of CD8^+^ T cells from chronically HIV-infected donors to upregulate these cytotoxic effector molecules following *in vitro* culture [18], and we have shown that CD8^+^ T cell cytotoxic potential, defined by the ability to rapidly upregulate perforin following brief stimulation *ex vivo*, correlates inversely with viral load [16].

Effector CD8^+^ T cell development is coordinated by an array of transcription factors [23]. Murine studies have identified the T-box transcription family members T-bet and eomesodermin (Eomes) as important regulators of the differentiation and function of cytotoxic effector T cells [24–26]. T-bet positively regulates genes associated with effector functions including perforin, granzyme B, and IFN-γ [27, 28], whereas Eomes is associated with the expression of perforin as well as proteins involved in maintenance of memory CD8^+^ T cells [24, 26, 29, 30]. While previous studies suggested a level of redundancy in the gene targets of these transcription factors, recent data show that the balance of T-bet and Eomes expression within a cell is a determinant of the differentiation pathway and functionality of the cell [30–34]. In the context of chronic HIV infection, HIV-specific CD8^+^ T cells with high levels of T-bet demonstrate greater overall functionality and maintain the ability to express perforin whereas cells with a T-bet^Lo^Eomes^Hi^ phenotype are less differentiated, less functional, exhausted, and express little to no perforin [28, 32]. Notably, during chronic progressive infection the T-bet^Lo^Eomes^Hi^ phenotype dominates the HIV-specific CD8^+^ T cell pool [32]. It remains unclear if low T-bet levels and the associated deficiency in perforin expression results from progressive loss on the part of responding HIV-specific CD8^+^ T cells or if responding cells are inherently dysfunctional throughout the infection period.

Much of our current knowledge regarding the dynamics of CD8^+^ T cell responses during acute infection is derived from murine models, particularly following infection with lymphocytic choriomeningitis virus, gammaherpesvirus, or influenza [35–37]. Infection by these viruses induces rapid and substantial activation and expansion of antigen-specific CD8^+^ T cells. Following resolution of acute viremia, the virus-specific population contracts, giving rise to memory cells that provide long-term protection. Human antiviral CD8^+^ T cell responses have primarily been assessed in the context of chronic infection, after the memory pool has been established [10, 38–41]. Recent studies have examined development of human CD8^+^ T cell responses to a range of primary infections, including attenuated yellow fever virus, attenuated vaccinia virus, influenza, tick-borne encephalitis virus (TBEV), hantavirus, and Epstein-Barr virus [42–47], demonstrating that antigen-specific cells have immediate cytotoxic capacity directly *ex vivo* during the acute phase of these infections. The few studies to examine the earliest responses to HIV showed that HIV-specific CD8^+^ T cells have limited functionality during the acute phase of infection but did not assess cytotoxic potential or regulation by T-bet or Eomes [48, 49], leaving the question unresolved as to whether these effector molecules are induced during acute infection.

Here, we examined the temporal dynamics of the CD8^+^ T cell effector response in peripheral blood of subjects experiencing acute primary HIV infection. We found that infection elicited a robust and highly activated response with immediate cytotoxic potential within the peripheral CD8^+^ T cell pool and that cells responding to short *in vitro* stimulation with HIV peptides were able to degranulate and rapidly upregulate perforin *de novo*. However, HIV-specific CD8^+^ T cells rapidly lost the ability to upregulate perforin following resolution of peak viremia. Loss of perforin expression coincided with a concurrent reduction in the expression of T-bet, but not Eomes, on a per-cell basis. Our data provide evidence of a robust and physiologically appropriate response during the earliest phase of acute HIV infection that is rapidly lost during progressive chronic infection, due in part to an inability to express sufficient levels of T-bet to properly drive effector differentiation.

## Results

### Acute HIV infection is associated with an expansion of the effector memory CD8^+^ T cell pool

**Fig 1:**
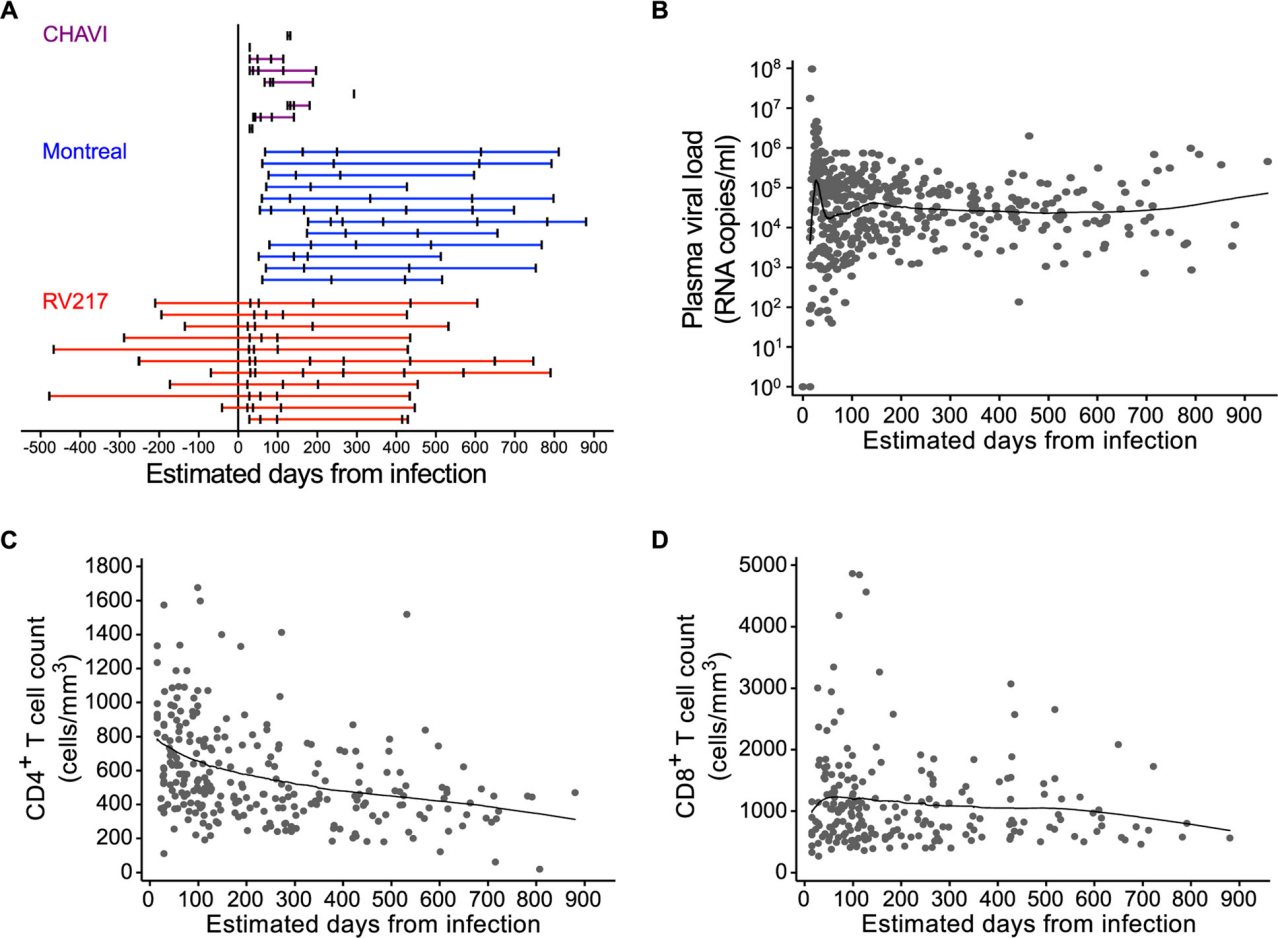
Timing of study participant samples and dynamics of HIV plasma viral loads, CD4^+^ T cell counts and CD8^+^ T cell counts. (**A**) Timing of samples relative to estimated time of infection for the three acute/early HIV cohorts: CHAVI (purple), Montreal (blue), and RV217 (red). Plasma HIV RNA copies/ml (**B**), absolute CD4^+^ T cells counts (**C**), and CD8^+^ T cell counts for all 32 donors (**D**). Lowess smoothers were used to represent the mean over time for longitudinal data.

Longitudinal samples were obtained from 32 subjects experiencing primary HIV infection (Fig 1A), 28 of whom had at least one acute time point (36 time points total; median 54 d from infection, range 23–100 d) and 23 with at least one chronic time point (40 time points total; median 551 d, range 367–880 d). Samples were drawn from three separate cohorts of acutely infected individuals: the CHAVI001 acute-infection cohort, the Montreal Primary Infection cohort, and the RV217/ECHO cohort. These cohorts provided broad geographical representation including North America, East Africa, Malawi, and Thailand (S1 Table). Subjects were antiretroviral therapy naïve at all time points, consistent with the standard of care at the time of study, and none controlled viral load to undetectable levels (Fig 1B). The mean peak viral load was 5.2 log_10_ RNA copies/ml for the entire study population (7.0 log_10_ RNA copies/ml for the better-characterized RV217 donors) and 4.42 log_10_ RNA copies/ml at set point. Peripheral blood CD4^+^ T cell counts and CD8^+^ T cell counts both declined over the study period (average rates of 80 cells/mm^3^ per year and 75 cells/mm^3^, respectively; Fig 1C and 1D). Samples from 41 seronegative healthy donors, including pre-infection time points for the 11 RV217 acute subjects (median -210 d from infection, range -41 to -478 d; Fig 1A and S1 Table), were analyzed for comparison.

**Fig 2:**
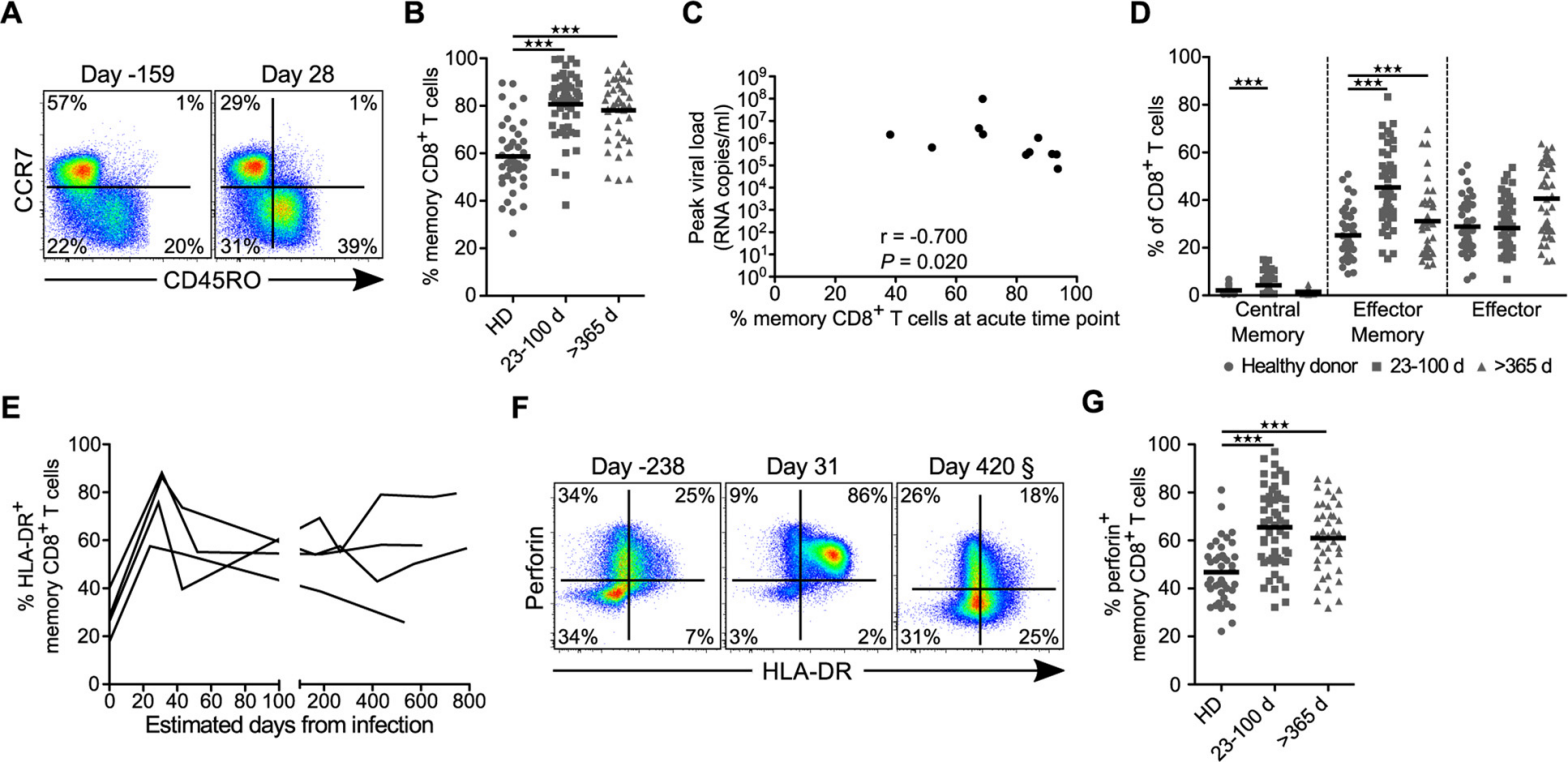
Memory distributions, activation, and proportion of cytotoxic peripheral CD8^+^ T cells for healthy donors and following HIV infection. (**A**) Representative flow cytometric plots of CCR7 versus CD45RO from a pre- (day -159) and acute (day 28) infection time point for one donor. (**B**) Proportion of circulating total memory CD8^+^ T cells for all healthy donors (HD; *n* = 41), acute HIV time points (23–100 d; *n* = 27), and chronic HIV time points (>365 d; *n* = 23). (**C**) Peak viral load plotted against total memory CD8^+^ T cells at the earliest available time point post-infection (23–41 d) for each RV217 donor. Spearman’s rank correlation test was used to determine significance. (**D**) Memory subsets as determined by CCR7 and CD45RO staining for all healthy donors (circles), acute HIV time points (squares), and chronic HIV time points (triangles). (**E**) Proportion of memory CD8^+^ T cells that express HLA-DR over time from infection for four RV217 subjects. Pre-infection time points were set as day 0 for analysis. (**F**) Representative flow cytometric plots of perforin and HLA-DR expression by memory CD8^+^ T cells from a pre- (day -173), acute (day 31), and chronic (day 420) infection time point for one donor. § Day 420 sample was acquired and analyzed at a later date than earlier samples resulting in a different gating scheme. For consistency, gates were set using naïve (CCR7^+^CD45RO^-^) CD8^+^ T cells, which generally do not express perforin or HLA-DR. (**G**) Proportion of memory CD8^+^ T cells that express perforin for healthy donors (HD), acute HIV time points (23–100 days), and chronic HIV time points (> 365 days). All data represent direct *ex vivo* assessment with no *in vitro* stimulation. *** denotes a *P* value < 0.001. Statistics based on a GEE population-averaged model with Holm adjusted *P* value. Bars represent approximations of the means generated by the models.

To determine if different phases of infection were associated with changes in circulating CD8^+^ T cell differentiation and activation, we assessed the size and composition of the memory CD8^+^ T cell pool (S1 Fig). Relative to HIV-negative donors, HIV-infected subjects had a significantly larger memory (non-CCR7^+^CD45RO^-^) CD8^+^ T cell pool in both the acute and chronic phases of infection (Fig 2A and 2B). Of note, the frequency of total memory CD8^+^ T cells at the earliest post-infection time points inversely correlated with peak viral load, but not with set point viral load (Figs 2C and S2A). In addition to the larger memory pool we also observed a shift in the distribution of memory subsets in infected subjects, with significantly higher proportions of central memory (CCR7^+^CD45RO^+^) and, predominately, effector memory (CCR7^-^CD45RO^-^) subsets during acute infection (Fig 2D). Only the effector memory pool remained significantly elevated into the chronic phase. There was no difference in the proportion of the effector cell pool (CCR7^-^CD45RO^-^) during either phase of infection, although the relative frequency of these cells did appear to be larger as infection progressed (Fig 2D).

When we examined the activation state of the memory pool for four RV217 subjects by measuring surface expression of HLA-DR, we found massive levels of activation within the memory CD8^+^ T cell compartment following HIV infection (Fig 2E), in agreement with recent data from Ndhlovu *et al*. [48]. To determine if this population of highly activated cells expressed cytolytic molecules directly *ex vivo* we measured perforin content. We found that almost all HLA-DR^+^ cells expressed perforin during the acute phase (Fig 2F). In addition, we observed a significantly greater proportion of perforin^+^ cells in both acute and chronic phases of infection compared to healthy donors (Fig 2G). There was, however, no significant association between the frequency of perforin^+^ CD8^+^ T cells and viral load at any time point (S2B Fig). Together, these data show that during acute HIV infection a large proportion of the peripheral CD8^+^ T cell pool is highly activated and primed to exert cytotoxic effector activity but the absolute magnitude of total cytotoxic CD8^+^ T cells does not predict set point viral load.

### HIV infection increases the total peripheral cytotoxic CD8^+^ T cell pool

We next examined if the large frequency of cytotoxic CD8^+^ T cells observed during acute HIV infection was consistent across other acute viral infections. We compared the total CD8^+^ T cell responses of subjects from the RV217 cohort with those of HIV-negative individuals who were vaccinated with attenuated vaccinia virus (VV) or attenuated yellow fever virus (YFV)-17D, or experimentally infected with a H1N1 strain of influenza virus (S3A–S3C Fig). Vaccination with VV or YFV elicits a robust and highly specific CD8^+^ T cell response that peaks approximately two weeks after inoculation and is largely resolved by four weeks [46]. The peripheral CD8^+^ T cell response to influenza is less robust, peaks at 1–2 weeks, and resolves by four weeks post-infection [43].

**Fig 3:**
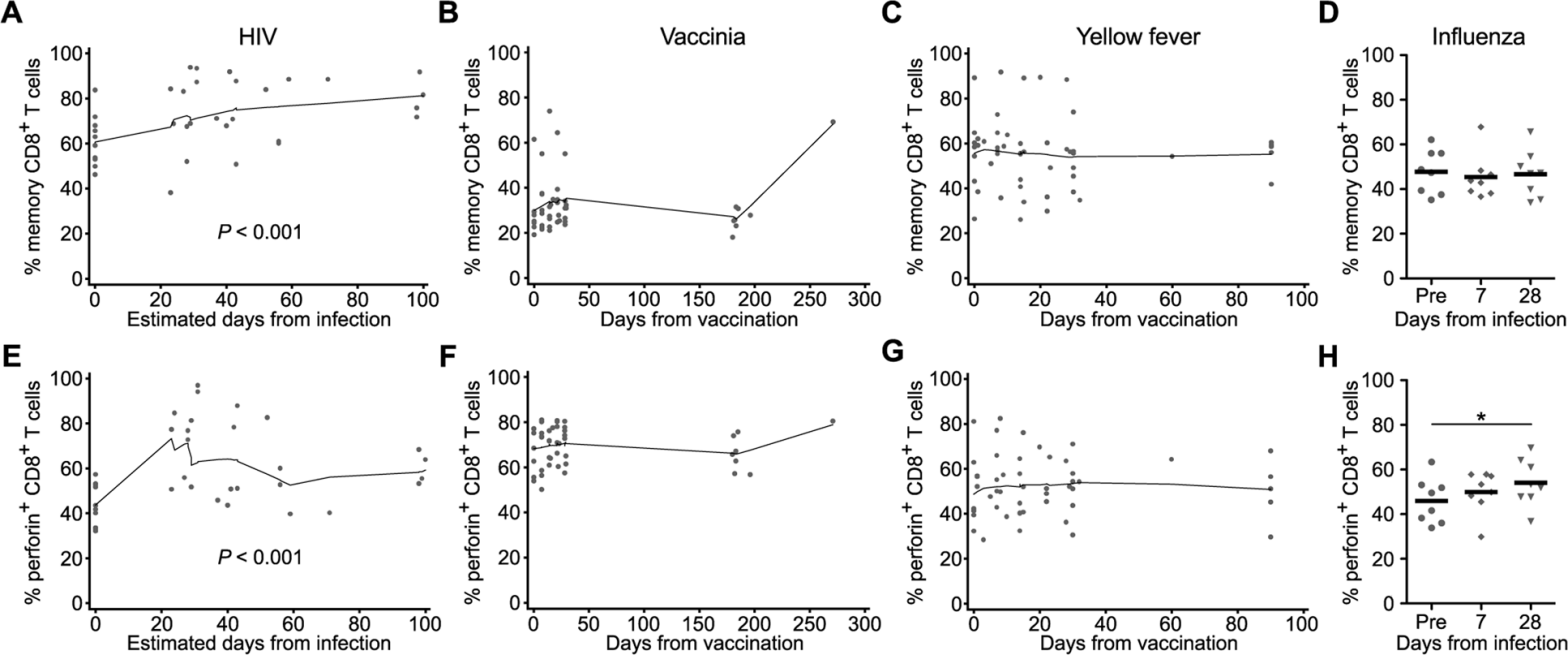
Dynamics of the total CD8+ T cell memory pool and cytotoxic response following infection with HIV, vaccinia virus, yellow fever virus, or influenza. Proportion of total memory CD8^+^ T cells for longitudinal time points from donors either naturally infected with HIV (*n* = 11; **A**), vaccinated with attenuated vaccinia virus (Dryvax, *n* = 8; **B**), vaccinated with live yellow fever virus (YFV-17D, *n* = 10; **C**), or experimentally infected with influenza (strain H1N1, *n* = 10; **D**). Frequency of memory CD8^+^ T cells that express perforin following infection with HIV (**E**), vaccinia (**F**), yellow fever (**G**) or influenza (**H**). Pre = pre-infection or pre-vaccination time points. Pre-infection time points for HIV, vaccinia, and YFV, were set as day 0 for analysis. All data represent direct *ex vivo* assessment with no *in vitro* stimulation. * denotes a *P* value < 0.05. Statistics based on a GEE population-averaged model with Holm adjusted *P* value. Bars represent approximations of the means generated by the models. Lowess smoothers were used to represent the mean over time for longitudinal data.

Consistent with the comparison between healthy donors and acute phase HIV infection (Fig 2B), both the total memory CD8^+^ T cell pool and the effector memory subset increased significantly from pre- to acute HIV infection (Figs 3A and S4E). There was also a significant increase in the proportion of perforin^+^ cells over the first thirty days of infection, with almost all (>90%) circulating memory CD8^+^ T cells expressing perforin in some donors (Fig 3E). When we examined the CD8^+^ T cell responses to *in vivo* stimulation following vaccination with VV or YFV, or infection with influenza, we did not observe significant changes in the size or distribution of the peripheral memory pool (Figs 3B–3D and S4). We did find increased levels of activated HLA-DR^+^ cells in some donors after vaccination with VV and YFV, but frequencies of perforin^+^ cells remained relatively stable throughout the entire vaccine course (Figs 3F, 3G and S5A–S5D). Only infection with influenza resulted in a slight but significant increase in perforin^+^ cells at d28 post-infection (Fig 3H). While these models of acute viral infections do have limitations in their use as comparators for our HIV-infected donors (e.g. different antigen loads, different localizations, and more precise timing of infection), overall these data show the dramatic increase in cytotoxic cells that takes place in the peripheral blood of HIV acutely infected subjects is significantly more pronounced compared to live-attenuated vaccination or influenza infection.

### HIV-specific CD8^+^ T cell cytotoxic capacity decreases as HIV infection progresses

**Fig 4:**
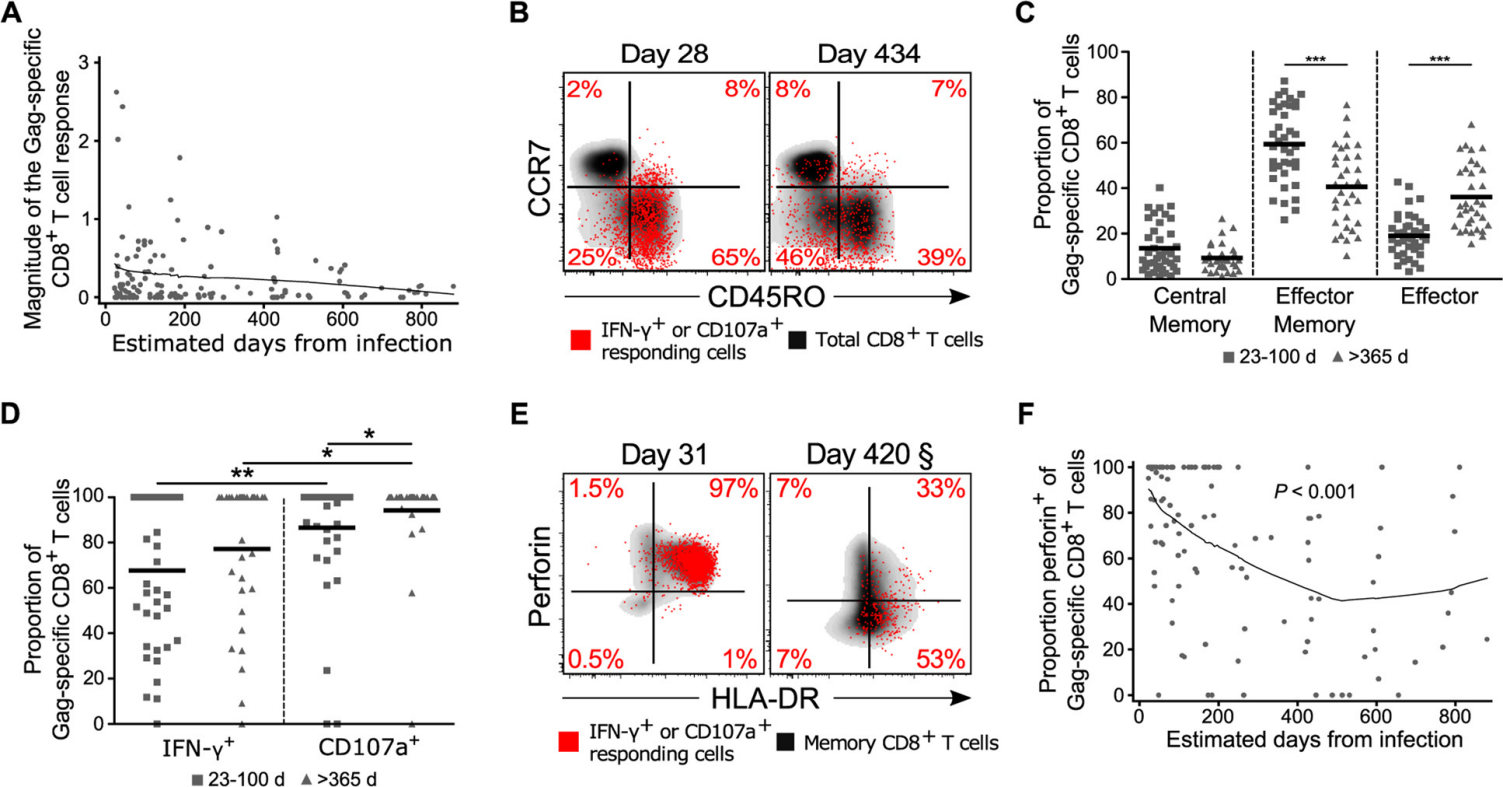
Magnitude and functionality of HIV-specific responses over time. (**A**) Frequency of Gag-specific CD8^+^ T cells within the memory CD8^+^ T cell pool over time as determined by measurement of IFN-γ expression or degranulation (CD107a) in response to peptide stimulation (*n* = 32). (**B**) Memory distributions for Gag-specific CD8^+^ T cells (red) overlaid on total CD8^+^ T cells (black) for a representative donor. (**C**) Memory distributions for responding Gag-specific CD8^+^ T cells as determined by CCR7 and CD45RO staining for acute (squares; *n* = 23), and chronic (triangles; *n* = 23) HIV time points. (**D**) Proportion of total responding Gag-specific CD8^+^ T cells that have upregulated IFN-γ or degranulated at acute and chronic time points. (**E**) Gag-specific CD8^+^ T cells (red) overlaid on total memory CD8^+^ T cells (black) for a representative donor. Percentages represent frequency of responding Gag-specific cells within a quadrant. § Day 420 sample was acquired and analyzed at a later date than earlier samples resulting in a different gating scheme. For consistency gates were set using naïve (CCR7^+^CD45RO^-^) CD8^+^ T cells. (**F**) Proportion of total responding Gag-specific CD8^+^ T cells that upregulated perforin in response to peptide stimulation (*n* = 28). * denotes a *P* value < 0.05 and ** denotes a *P* value < 0.01. Statistics based on a GEE population-averaged model with Holm adjusted *P* value or random-effects tobit regression. Bars represent approximations of the means generated by the models. Lowess smoothers were used to represent the mean over time for longitudinal data.

We next sought to determine if the cytotoxic potential of HIV-specific cells demonstrated similar dynamics to the total memory CD8^+^ T cell pool during acute to chronic HIV infection. To identify HIV-specific cells we focused on the detection of IFN-γ production and CD107a-marked degranulation following a short-term *in vitro* stimulation with peptides derived from the HIV-1 Gag and Nef proteins [15, 48–50]. In agreement with previous studies that evaluated HIV-specific cells longitudinally by functional responses or tetramer staining [49, 51], we found no difference in the absolute magnitude of responding cells for either protein over time (Figs 4A and S6A). Consistent with the memory distribution of the total CD8^+^ T cell pool, Gag-specific cells largely had an effector memory phenotype in the acute phase of infection but became more equally distributed between effector and effector memory subsets for early chronic time points (Fig 4B and 4C). Also in agreement with previous data, cells tended to degranulate more readily than upregulate IFN-γ in the acute phase of infection (Figs 4D and S6B)[48, 49]. The high proportion of degranulating cells suggested that the HIV-specific response might be cytotoxic over the course of infection, as analysis of the total CD8^+^ T cell pool had indicated. However, degranulation is not an absolute surrogate of cytolytic potential [16, 52], nor does it indicate whether the cells will continue to be cytotoxic following the initial granule release [53]. To assess cytotoxic potential more directly, we measured perforin expression levels within the Gag- and Nef- specific cells (Figs 4E and S6C). The majority of cells that responded to direct *ex vivo* stimulation rapidly upregulated perforin during the earliest time points following infection, suggesting that the early HIV-specific response was likely highly cytotoxic. In contrast to the bulk memory CD8^+^ T cell pool, however, as acute viremia was resolved there was a rapid loss of perforin expression by both HIV-1 Gag- and Nef-specific CD8^+^ T cells (Figs 4F and S6D).

A large proportion of HIV-specific CD8^+^ T cells have previously been shown to upregulate β-chemokines independently of degranulation during acute HIV infection [49]. To determine if β-chemokine-producing cells similarly expressed perforin, we assessed expression of MIP-1α by responding cells in a subset of subjects. Inclusion of MIP-1α did not significantly change the overall magnitude of Gag-specific cells detected over time, though it did identify a subset of cells not captured by IFN-γ or CD107a (S7A–S7C Fig). Importantly, the dynamics with which expression of perforin by Gag-specific cells was lost was the same with or without MIP-1α (S7D and S7E Fig). Combined, these data show similarities in the total and Gag-specific CD8^+^ T cell responses in both differentiation state and cytotoxic potential, suggesting the bulk of activated cells during acute HIV infection could be comprised of HIV-specific CD8^+^ T cells.

### Dissociation of T-bet and Eomes expression from cytolytic potential in memory CD8^+^ T cells in acute HIV infection

**Fig 5:**
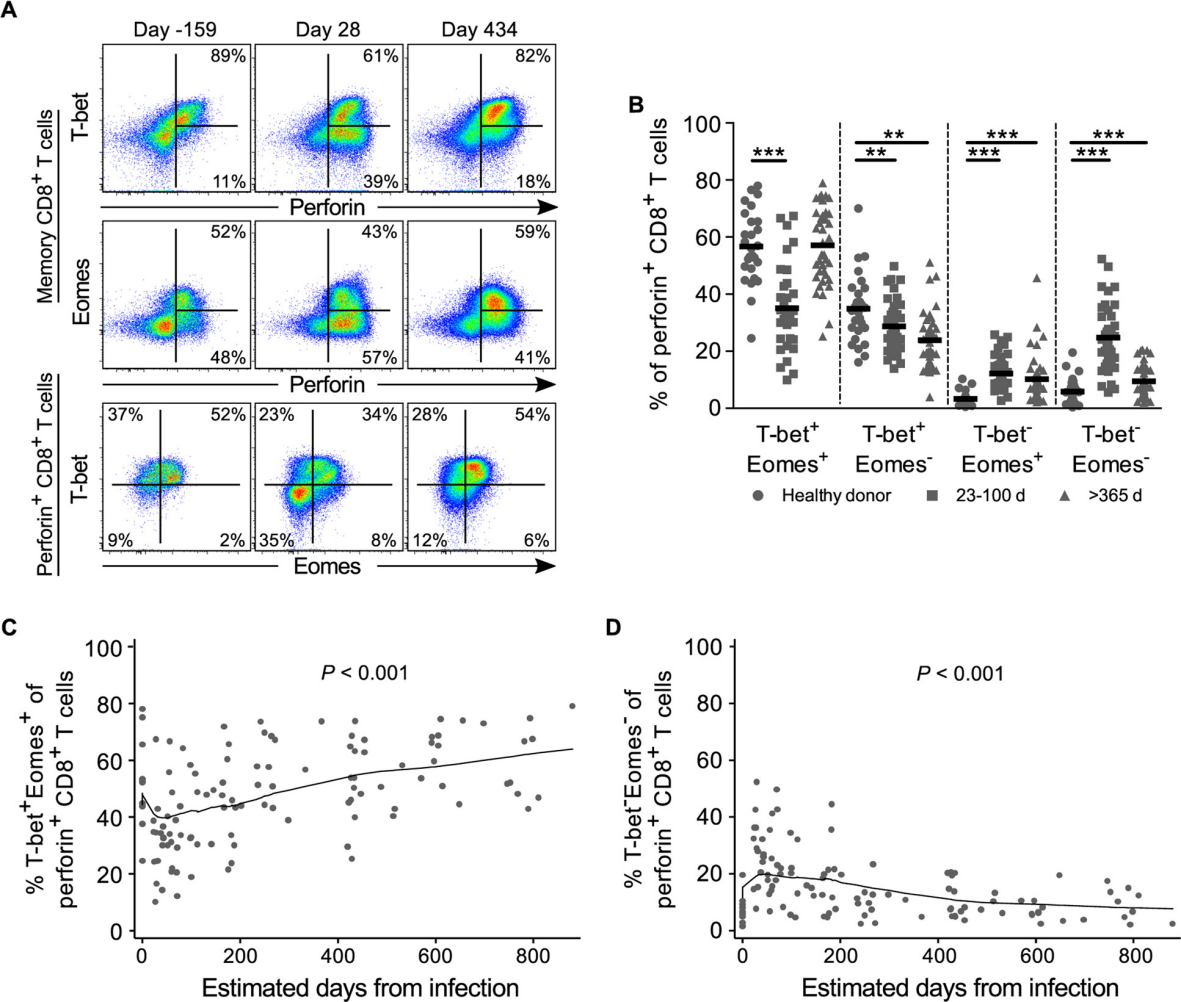
T-bet and Eomes expression by total perforin^+^ CD8^+^ T cells for healthy donors and following HIV infection. (**A**) Representative flow cytometric plots of T-bet and Eomes expression for total memory (top and middle rows) and perforin^+^ (bottom row) CD8^+^ T cells from the pre- (day -159), acute (day 28), and chronic (day 434) infection time points for one donor. Percentages on top and middle plots are of perforin^+^ cells within the total memory pool. (**B**) T-bet and Eomes expression by perforin^+^ CD8^+^ T cells for all healthy donors (circles; *n* = 29), acute HIV time points (squares; *n* = 21), and chronic HIV time points (triangles; *n* = 23). Frequency of perforin^+^ cells that are T-bet^+^Eomes^+^ (**C**) or T-bet^-^Eomes^-^ (**D**) over time (*n* = 23). All data represent direct *ex vivo* assessment with no *in vitro* stimulation. ** denotes a *P* value < 0.01 and *** denotes a *P* value < 0.001. Statistics based on a GEE population-averaged model with Holm adjusted *P* value. Bars represent approximations of the means generated by the models. Lowess smoothers were used to represent the mean over time for longitudinal data.

Studies in both murine models and humans have strongly linked the transcription factors T-bet and Eomes to the regulation of effector CD8^+^ T cell differentiation and function, including the expression of perforin [24–26, 28, 30, 42, 54]. To gain further insight into the evolution of the cytotoxic CD8^+^ T cell response to HIV we assessed the expression of T-bet and Eomes over the course of infection. For healthy donors, including HIV pre-infection time points, perforin expression was directly associated with T-bet and/or Eomes expression such that the majority of perforin^+^ cells were either T-bet^+^Eomes^+^ or T-bet^+^Eomes^-^ (Fig 5A and 5B). In contrast, acutely HIV-infected individuals showed marked dissociation between perforin and both T-bet and Eomes resulting in significantly lower proportions of T-bet^+^Eomes^+^ and T-bet^+^Eomes^-^ perforin^+^ cells (Fig 5A and 5B), and an expansion of perforin^+^ cells expressing neither T-bet nor Eomes. By the chronic stage these subsets had largely, though incompletely, returned to their normal distributions. When we analyzed T-bet and Eomes expression longitudinally for perforin^+^ CD8^+^ T cells within the HIV-infected cohort we found the proportion of T-bet^+^Eomes^+^ cells decreased over the first 30 days of infection and T-bet^-^Eomes^-^ cells increased over the first 60 days before gradually returning to pre-infection levels (Fig 5C and 5D). We have previously shown that the level of T-bet expression within peripheral CD8^+^ T cells is directly associated with perforin expression, where perforin was found predominantly within T-bet^Hi^ cells [28]. Consistent with those findings, perforin was most highly associated with a T-bet^Hi^Eomes^+^ expression pattern in HIV negative donors and this subset experienced the largest drop during acute HIV (S8 Fig). Despite these shifts in expression patterns that appeared to coincide with the rise and fall plasma viremia, there was no association between the acute frequencies of T-bet or Eomes subsets and acute or set point viral loads (S9A–S9D and S10A–S10D Figs). However, frequencies of T-bet^+^ and T-bet^-^Eomes^-^ CD8^+^ T cells at set point time points were inversely or directly associated with set point viral load, respectively (S9A and S9D Fig).

To determine if the dissociation between perforin, T-bet, and Eomes was unique to HIV, we examined T-bet and Eomes expression within total perforin^+^ cells following YFV and VV vaccination. While we found almost no dissociation for YFV, there was a transient dissociation following vaccination with vaccinia, although not to the same extent as observed during acute HIV (S11A and S11B Fig). We next examined expression of T-bet and Eomes within HLA-DR^+^ cells throughout the different vaccine courses. As noted above, during acute HIV infection the vast majority of HLA-DR^+^ cells are also perforin^+^ (Fig 2E); thus, it was unsurprising to find that perforin^+^ and HLA-DR^+^ cells showed almost identical dynamics in the loss of T-bet and Eomes expression for HIV (S11C Fig). Similarly, for both YFV and VV, activated cells showed a transient increase in the frequency of T-bet^-^Eomes^-^ cells at day 14 post-vaccination. Together these data suggest that the transient expansion of highly activated bulk effector CD8^+^ T cells during acute viral infection in humans may not require expression and/or maintenance of T-bet and Eomes.

### HIV-specific cells retain T-bet and Eomes expression, but maintenance of cytotoxic potential over time is associated with higher per-cell T-bet levels

**Fig 6:**
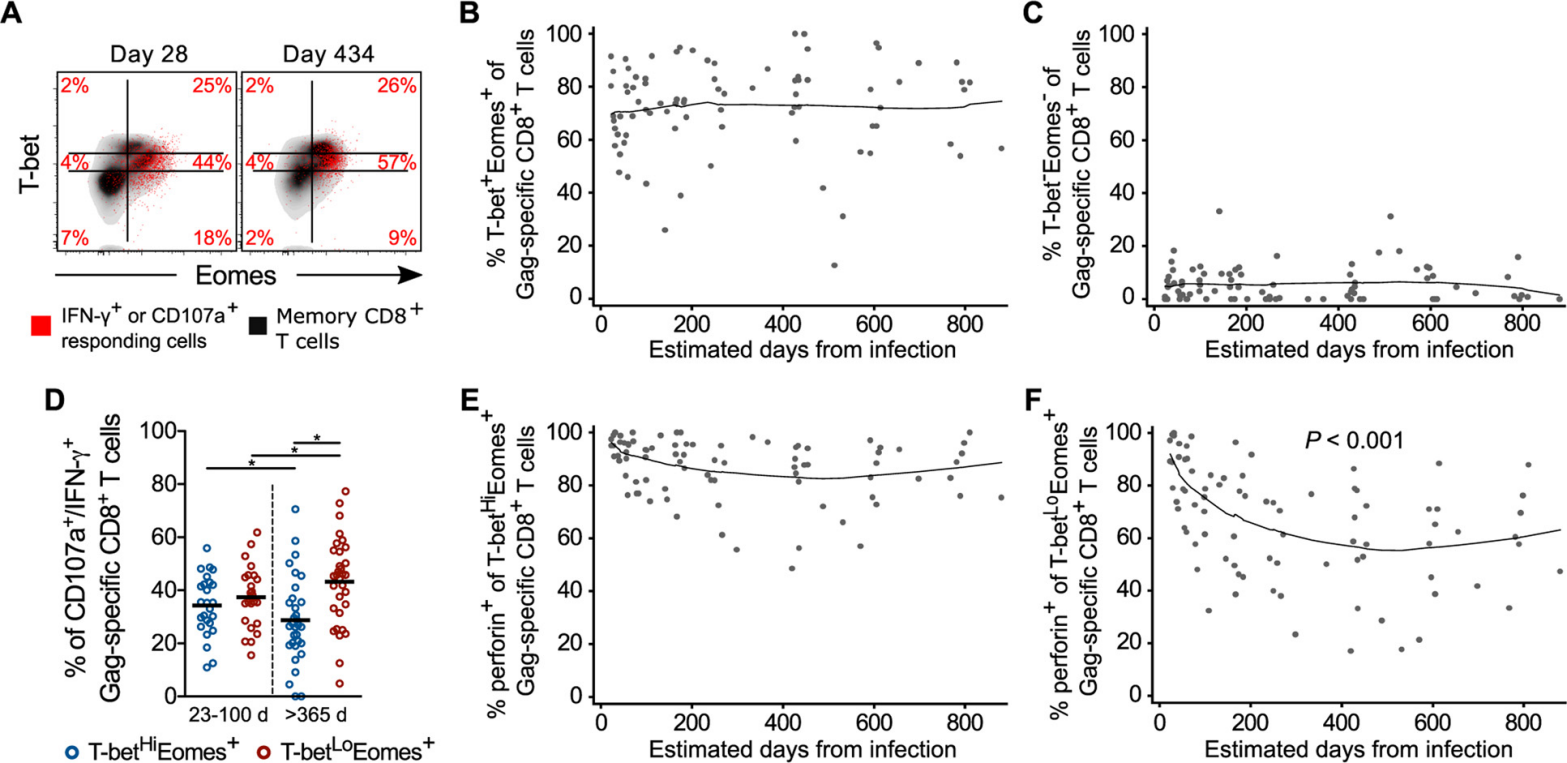
T-bet and Eomes expression by responding HIV-specific CD8^+^ T cells over time. (**A**) Gag-specific CD8^+^ T cells (red) overlaid on perforin^+^ CD8^+^ T cells (black) from the acute (day 28) and chronic (day 434) infection time points of one donor. Percentages of responding Gag-specific cells within each T-bet and Eomes subset are provided. Frequencies of T-bet^+^Eomes^+^ (**B**) and T-bet^-^Eomes^-^ (**C**) responding Gag-specific CD8^+^ T cells over time. (**D**) Frequencies of T-bet^Hi^Eomes^+^ (blue circles) and T-bet^Lo^Eomes^+^ (red circles) responding Gag-specific CD8^+^ T cells at acute (23–100 days; *n* = 16) and chronic (>365 days; *n* = 23) time points. Proportion of T-bet^Hi^Eomes^+^ (**E**) and T-bet^Lo^Eomes^+^ (**F**) responding Gag-specific CD8^+^ T cell subsets that express perforin over time. *n* = 20 for all longitudinal graphs. * denotes a *P* value < 0.05. Statistics based on a GEE population-averaged model with Holm adjusted *P* value or random-effects tobit regression. Bars represent approximations of the means generated by the models. Lowess smoothers were used to represent the mean over time for longitudinal data.

To determine if the transient loss of T-bet and Eomes within the bulk activated CD8^+^ T cell memory pool during acute HIV infection extended to HIV-specific CD8^+^ T cells, we assessed expression of these transcription factors in Gag-specific CD8^+^ T cells. In marked contrast to the highly activated bulk CD8^+^ T cell effector population during acute HIV infection, HIV-specific CD8^+^ T cells expressed T-bet and/or Eomes at the earliest detectable time point and throughout the course of infection (Fig 6A–6C). This indicates that despite their phenotypic similarities total and HIV-specific CD8^+^ T cells may be primed quite differently during acute infection and raises the possibility that the majority of expanded effector CD8^+^ T cells in early HIV infection may not be specific for HIV.

We next examined whether loss of perforin expression was related to changes in the level of T-bet expression during early HIV infection. Interestingly, the distribution of T-bet within Gag-specific CD8^+^ T cells changed over time from acute to chronic infection (Fig 6D). In the acute phase, responding cells were equally distributed between T-bet^Hi^Eomes^+^ and T-bet^Lo^Eomes^+^ expression patterns, which during the chronic phase began to be dominated by T-bet^Lo^Eomes^+^ cells (Fig 6D). Furthermore, T-bet^Hi^Eomes^+^ HIV-specific CD8^+^ T cells continued to express perforin as infection progressed, whereas T-bet^Lo^Eomes^+^ cells gradually lost perforin expression over time (Fig 6E and 6F).

Finally, in contrast to the recent findings by Ndholuvu, et al. [48], we did not find the magnitude, proportion perforin^+^, or any T-bet- or Eomes-expressing subset of responding HIV-1 Gag-specific CD8^+^ T cells to be predictive of peak or set point viral load (S12 and S13 Figs). Despite this, our data suggest that in the earliest phase of infection, HIV-specific CD8^+^ T cells have both the transcriptional and functional properties associated with long-term control of HIV replication [16, 28], and that the inability to durably maintain high-level T-bet expression contributes to a qualitatively inferior response as infection progresses.

## Discussion

Mechanisms underlying the inability of CD8^+^ T cells to fully control HIV replication have remained unclear. Failure of antiviral immunity has been attributed in part to qualitative defects in total and HIV-specific CD8^+^ T cells [15, 16, 20, 55, 56]. However, the dysfunction observed within the CD8^+^ T cell pool has largely been defined in the context of chronic infection when the success or failure of the presumed response has already been determined. The question of whether CD8^+^ T cells in progressive infection were intrinsically less functional from the outset or if dysfunction arose over time has remained unanswered. To address this issue, we assessed the longitudinal CD8^+^ T cell responses of a diverse cohort of individuals experiencing acute/early HIV infection. We show that acute HIV infection elicits a robust cytotoxic CD8^+^ T cell response characterized by cells that express the cytolytic effector molecule perforin and the effector-associated transcription factors T-bet and Eomes. Importantly, the quality of the response quickly waned following the resolution of acute viremia, with a significant decrease in perforin expression by HIV-specific CD8^+^ T cells that was at least partially accounted for by a shift from T-bet^Hi^Eomes^+^ cells to T-bet^Lo^Eomes^+^ cells. The attenuation of the cytolytic response may help explain the failure of CD8^+^ T cells to control HIV replication in the long-term.

It is well documented that CD8^+^ T cell responses are elicited early in HIV infection and are associated with control of viral replication [1, 2, 48, 57]. Some of the strongest evidence of the CD8^+^ T cell-mediated immunologic pressure exerted during this period is the rapid emergence of viral escape mutations within known CD8^+^ T cell epitopes [4, 6, 9]. We found that HIV-specific cells had high cytotoxic potential at the earliest time points following HIV infection, but rapidly lost this function as disease progressed. This suggests a mechanism through which CD8^+^ T cells may exert a strong direct selective pressure on the virus resulting in the rapid selection of escape variants early in infection that ultimately have a reduced capacity to stimulate cytolytic CD8^+^ T cell responses [6, 9, 58, 59]. It should be noted that whereas perforin expression was lost over time almost all HIV-specific responding cells continued to produce MIP-1α. Thus, while cytotoxic CD8^+^ T cells play an important role in the resolution of acute viremia, as they lose their ability to express perforin they may be able to keep the virus partially in check through a combination of the remaining cytotoxic response and non-cytotoxic inhibitory effects exerted via the continued expression of β-chemokines or other non-cytolytic mechanisms [60]. This would be consistent with models suggesting CD8+ T cell cytotoxic mechanisms do not account for the entirety of CD8^+^ T cell-mediated viral suppression during chronic progressive SIV infection [61, 62]. It remains unclear if maintenance of perforin expression following acute infection would further enhance the level of control over viral replication CD8^+^ T cells provide as we would predict it should based on studies of CD8^+^ T cell responses in the chronic phase of infection [16, 18, 19]. Unfortunately, we were unable to find any direct associations between HIV-1 Gag-specific perforin, T-bet, or Eomes expression and the level of plasma viremia or CD4^+^ T cell numbers.

T-bet and Eomes are important regulators of effector CD8^+^ T cell differentiation and function for both mice and humans [24–26, 28, 30, 31, 33, 34, 42, 54]. Expression patterns of these transcription factors have been described for CD8^+^ T cells in the context of various human viral infections, including CMV, EBV, HBV, HCV, HIV, and TBEV [28, 32, 34, 42, 54, 63–66]. These studies demonstrated a high degree of variability in the relative levels of T-bet and Eomes expressed by virus-specific CD8^+^ T cells depending on time from infection, whether the infection was controlled, and tissue localization. CMV-specific cells express T-bet and Eomes during both acute and chronic phases of infection, but control of viral replication in the acute phase is associated with a higher ratio of T-bet^+^ versus Eomes^+^ cells [64, 66]. EBV- and TBEV-specific cells also express T-bet and Eomes during the earliest phase of their respective infections, but EBV-specific cells lose expression of both during convalescence whereas TBEV-specific cells retain T-bet expression and show a gradual reduction in Eomes [42, 63]. HCV-specific cells are T-bet^+^ in acute/resolving HCV infection and T-bet^-^Eomes^-^ during acute/non-resolving infection. Post-acute phase, HCV-specific cells in the peripheral blood are T-bet^-^Eomes^-^ for both resolved and non-resolved HCV infection, but T-bet^+^ within the livers of subjects with resolved infection and Eomes^+^ in livers of chronically infected subjects [34, 65]. Together, these results suggest expression of T-bet during the acute phase is a critical determinant of viral infection outcome. The differential outcomes associated with Eomes were also reflective of the relative expression level of T-bet, suggesting Eomes may not be as important for the resolution of acute viremia. Rather, Eomes expression may determine whether antigen-specific cells are fated to form a stable memory pool or become exhausted subsequent to the acute phase, dependent on whether or not the infection is ultimately cleared [34, 67].

Similar associations between T-bet, Eomes, and outcome have been demonstrated in chronic HIV infection. In this context, a high level of T-bet expression was associated with greater overall functionality of HIV-specific CD8^+^ T cells, including cytotoxic potential, and relative control of viral replication, whereas low T-bet levels and continued Eomes expression has been associated with lower overall functionality and persistent viremia [28, 32]. Our data show that HIV-specific cells have high cytotoxic potential during acute infection, but lose the ability to express or rapidly upregulate perforin in chronic infection. This loss of cytotoxic potential over time can at least partially be explained by a change in the relative expression levels of T-bet and Eomes: HIV-specific cells were equally T-bet^Hi^Eomes^+^ and T-bet^Lo^Eomes^+^ during acute infection and both subsets efficiently upregulated perforin initially but the proportion of T-bet^Lo^Eomes^+^ cells increased significantly as infection progressed and cells with this phenotype had an inferior capacity to express perforin compared to T-bet^Hi^Eomes^+^ cells. The expression of perforin by either phenotype during acute infection may be reflective of the high degree of inflammation and activation during this phase, a differential role for Eomes at different stages of infection, and/or the result of additional transcription factors not assessed here. Whatever the case may be, T-bet^Hi^Eomes^+^ HIV-specific CD8^+^ T cells retain the ability to upregulate perforin following resolution of acute viremia and this subset declines during chronic progressive infection.

Recent data from Ndhlovu *et al*. suggests HIV infection elicits a massive antigen-specific CD8^+^ T cell response with limited bystander activation [48]. Similar observations have been reported after vaccination with vaccinia and yellow fever virus [46]. The similarities in differentiation state, activation, and immediate cytotoxic potential between total peripheral memory and Gag-specific cells reported here support the idea of a robust and specific response to HIV infection. However, we found a significant discrepancy between transcriptional control of HIV-specific CD8^+^ T cells versus the bulk activated perforin^+^ memory CD8^+^ T cell population. The degree to which these differences reflect a true lack of specificity, dysfunction on the part of the bulk activated cells, an inability to identify an appropriate functional marker, or an attempt by the host to mitigate immune-mediated pathology remains unclear. It is likely there area many more circulating HIV-specific CD8^+^ T cells than indicated by our findings using *in vitro* stimulation with only two HIV-1 proteins and a limited number of functional parameters to identify responding cells. However, it should be noted that CD8^+^ T cell bystander activation has been reported during acute HIV and EBV infection in humans and it is possible at least a subset of CD8^+^ T cells are activated non-specifically in our cohort [44, 68]. T cell receptor stimulation is required for upregulation of T-bet [69], but a large proportion of bulk activated perforin^+^ cells during acute HIV infection appear to express neither T-bet nor Eomes whereas all Gag-specific cells expressed one or the other. In addition, perforin can be upregulated in the absence of direct antigenic stimulation via exposure to IFN-α [70], levels of which are highly elevated during acute HIV infection [71]. Thus, the difference in T-bet and Eomes expression we observed between bulk perforin^+^ and responding HIV-specific CD8^+^ T cells raises the possibility that a significant number of bystander-activated cells are being induced in response to HIV infection. Alternatively, given the association between activation and the size of the T-bet^-^Eomes^-^ pool across infections with vaccinia, yellow fever, and HIV, the absence of T-bet and Eomes expression in the bulk perforin^+^ CD8^+^ T cell pool may be a characteristic of the contraction phase that typically follows the initial CD8^+^ T cell response. This would be consistent with the pro-apoptotic phenotype of the majority of cells following peak HIV viremia and the timing of our samples [48]. Whether HIV-specific or bystander, the lack of T-bet and Eomes expression by these cells suggests they would be unable to sustain perforin expression upon encountering infected target cells. This may in part explain the inability of bulk peripheral CD8^+^ T cells from acutely HIV infected individuals to efficiently inhibit viral replication *in vitro* and further suggests they would not make a meaningful contribution to long-term control of viral replication *in vivo* [72, 73].

These data show how the peripheral CD8^+^ T cell response to HIV evolves over the course of progressive infection. HIV-specific CD8^+^ T cells are able to upregulate perforin and T-bet initially but begin to lose this capacity soon after peak viremia, demonstrating for the first time that there is not an initial intrinsic inability of HIV-specific CD8^+^ T cells to upregulate these molecules. It remains unclear how or if these responses differ from those of CD8^+^ T cells from subjects who go on to spontaneously control viral replication to very low levels in the chronic phase. While we did find frequencies of T-bet^+^ and T-bet^-^Eomes^-^ total memory CD8^+^ T cells at set point time points were inversely or directly associated with set point viral load, respectively, we did not find any associations between viral load and the size of the total peripheral perforin^+^ pool or the magnitude or cytotoxic potential of HIV-1 Gag-specific cells at any time point. Nor did we find any subset of total memory or Gag-specific cells to be predictive of set point viral load for this group of subjects, possibly due to the limited number of very early time points and relatively narrow range of viral loads at set point. However, the fact that the initial phenotype of HIV-specific cells is similar to that associated with control during the chronic phase of infection suggests induction and maintenance of cells capable of upregulating high levels of T-bet and perforin could lead to subsequent control. Eliciting HIV-specific cells with these characteristics might serve as an important target for vaccination or therapeutic modalities seeking to fully control early viral replication or eradicate the chronic viral reservoir.

## Materials and Methods

### Ethics statement

Blood specimens were acquired with the written informed consent of all study participants and with the approval of the institutional review board at each respective institution where patient materials were collected: University of Pennsylvania (IRB# 809316), McGill University Health Centre (REB# GEN-10-084), Human Subjects Protection Branch (RV217/WRAIR#1373), Kenya Medical Research Council (KEMRI/RES/7/3/1), The United Republic of Tanzania Ministry of Health and Social Welfare (MRH/R.10/18/VOLL.VI/85), Tanzanian National Institute for Medical Research (NIMR/HQ/R.8aVol.1/2013), Royal Thai Army Medical Department (IRBRTA 1810/2558), Uganda National Council for Science and Technology–National HIV/AIDS Research Committee (ARC 084), Uganda National Council of Science and Technology (HS 688), East London and City and the Southwest and Southwest Hampshire Ethics Review Committees, Duke University (IRB# Pro00006579 and IRB# Pro00007558), Emory University (IRB# 00009560), Oregon Health and Science University (IRB# 2470 and IRB# 2832). The study was conducted in accordance with the principles expressed in the Declaration of Helsinki.

### Study participants

Eleven HIV-1 acutely infected participants were enrolled as part of the RV217 Early Capture HIV cohort, nine were enrolled in the CHAVI 001 acute infection cohort, and twelve were enrolled in the Montreal Primary Infection cohort. Participant demographics are summarized in S1 Table. Acute HIV-1 infection was determined by measuring plasma HIV RNA content and HIV-specific antibodies using ELISA and Western blot. Fiebig staging [74] immediately following the first positive visit or at the screening visit was used to characterize the timing of infection for RV217 and CHAVI participants, respectively. The only exception was RV217 donor 40067 for which the estimated date of infection was taken as the midpoint between the last negative and first positive visit. For the Montreal Primary Infection cohort the following guidelines proposed by the Acute HIV Infection Early Disease Research Program sponsored by the National Institutes of Health were used to estimate the date of infection: the date of a positive HIV RNA test or p24 antigen assay available on the same day as a negative HIV enzyme immunoassay (EIA) test minus 14 days; or the date of the first intermediate Western blot minus 35 days. In addition, information obtained from questionnaires addressing the timing of high-risk behavior for HIV transmission was taken into account in assigning a date of infection when consistent with biological tests. The timing of visits relative to estimated date of infection for all acutely HIV infected donors used in this study is provided in Fig 1A. Study participants were antiretroviral therapy naïve at all time points analyzed, consistent with the standard of care at the time of study. HIV-1 viral loads were measured using the Abbot Real-Time HIV-1 assay (RV217; Abbot Laboratories, Abbott Park, IL), COBAS AMPLICOR HIV-1 monitor test, version 1.5 (CHAVI; Roche Diagnostics, Branchburg, NJ), or the UltraDirect Monitor assay (Montreal; Roche Diagnostics, Branchburg, NJ). HIV set point viral loads were defined as the average of all viral load measurements between 90 and 365 days post-infection in the absence of therapy with the requirement for at least two viral load measurements during this period.

For HIV-negative cohorts, volunteers were administered the live-attenuated YFV-17D vaccine (YF-Vax, Sanofi Pasteur), the live vaccinia smallpox vaccine (Dryvax, Wyeth Laboratories), or challenged with influenza A/Brisbane/59/07. YF-Vax was administered subcutaneously in the arm, Dryvax was administered by scarification of the upper arm with three pricks of a bifurcated needle, and influenza A virus was administered intra-nasally. Peripheral blood mononuclear cells (PBMCs) from pre-vaccination or pre-infection time points were available for most donors along with several time points post-vaccination or infection (S3A–S3C Fig). Pre-infection time points from all cohorts, including RV217 participants, along with PBMCs obtained from fifteen healthy human subjects through the University of Pennsylvania’s Human Immunology Core were combined for a total of 41 healthy donor data points.

### Peptides

Potential T cell epitope (PTE) peptides corresponding to the HIV-1 Gag and Nef proteins were obtained from the NIH AIDS Reagent Program (NIH, Bethesda, Maryland, USA). PTE peptides are 15 amino acids in length and contain naturally occurring 9 amino acid sequences that are potential T cell determinants embedded in the sequences of circulating HIV-1 strains worldwide, including subtypes A, B, C, D and circulating recombinant forms (CRF). As such, these peptide pools provided the coverage necessary for the T cell stimulation assays performed in this study given the broad geographical distribution of our study participants and diversity of infecting viruses (S1 Table). Lyophilized peptides were dissolved in dimethyl sulfoxide (DMSO, Sigma-Aldrich, St Louis/Missouri, USA), combined into two pools at 400 μg/ml, and stored at -20°C.

### PBMC stimulation

Cryopreserved PBMCs were thawed and rested overnight at 2x10^6^ cells/ml in RPMI medium supplemented with 10% fetal bovine serum, 2 mM L-glutamine, 100 U/ml penicillin, and 100 mg/ml streptomycin. Cell viability was checked both immediately after thawing and after overnight rest by trypan blue exclusion. Costimulatory antibodies (anti-CD28 and anti-CD49d, 1 μg/mL each; BD Biosciences) and pre-titrated fluorophore conjugated anti-CD107a was included at the start of all stimulations. PBMCs were incubated for 1 hour at 37°C and 5% CO_2_ prior to the addition of monensin (1 μg/mL; BD Biosciences) and brefeldin A (10 μg/mL; Sigma-Aldrich) followed by an additional 5 hour incubation at 37°C and 5% CO_2_. For peptide stimulations, peptides from the two Gag PTE pools were added to a single tube of cells such that each individual peptide was at a final concentration of 1 μg/ml. As a negative control, DMSO was added to the cells at an equivalent concentration to the one used for peptide stimulation.

### Antibody reagents

Antibodies for surface staining included CCR7 APC-Cy7 (clone G043H7; Biolegend), CCR7 APC-eFluor780 (clone 3D12; eBioscience), CD4 PE-Cy5.5 (clone S3.5; Invitrogen), CD8 BV711 (clone RPA-T8; Biolegend), CD8 Qdot 605 (clone 3B5; Invitrogen), CD14 BV510 (clone M5E2; Biolegend), CD14 Pacific Blue (clone M5E2; custom), CD14 PE-Cy5 (clone 61D3; Abcam), CD14 PE-Cy7 (clone HCD14; Biolegend), CD16 Pacific Blue (clone 3G8; custom), CD16 PE-Cy5 (clone 3G8; Biolegend), CD16 PE-Cy7 (clone 3G8; Biolegend), CD19 BV510 (clone HIB19; Biolegend), CD19 Pacific Blue (clone HIB19; custom), CD19 PE-Cy5 (clone HIB19; Biolegend), CD19 PE-Cy7 (clone HIB19; Invitrogen), CD45RO ECD (clone UCHL1; Beckman Coulter), CD45RO PE-CF594 (clone UCHL1; BD Biosciences), CD107a PE-Cy5 (clone eBioH4A3; eBioscience), CD107a PE-Cy7 (clone H4A3; Biolegend), and HLA-DR Pacific Blue (clone LN3; Invitrogen). Antibodies for intracellular staining included CD3 BV570 (clone UCHT1; Biolegend), CD3 BV650 (clone OKT3; Biolegend), CD3 Qdot 585 (clone OKT3; custom), CD3 Qdot 650 (clone S4.1; Invitrogen), Eomes Alexa 647 (WD1928; eBioscience), Eomes eFluor 660 (WD1928; eBioscience), IFN-γ Alexa 700 (clone B27; Invitrogen), Perforin BV421 (clone B-D48, Biolegend), Perforin Pacific Blue (clone B-D48; custom), Perforin PE (clone B-D48, Cell Sciences), T-bet FITC (clone 4B10; Biolegend), and T-bet PE (clone 4B10; eBioscience).

### Flow cytometric analysis

At the end of the stimulations, cells were washed once with PBS prior to be being stained for CCR7 expression for 15 min at 37°C in the dark. Cells were then stained for viability with aqua amine-reactive viability dye (Invitrogen) for 10 min at room temperature in the dark followed by addition of a cocktail of antibodies to stain for surface markers for an additional 20 min. The cells were washed with PBS containing 0.1% sodium azide and 1% BSA, fixed and permeabilized using a Cytofix/Cytoperm kit (BD Biosciences), and stained with a cocktail of antibodies against intracellular markers for 1 h at room temperature in the dark. The cells were washed once with Perm Wash buffer (BD Biosciences) and fixed with PBS containing 1% paraformaldehyde. Fixed cells were stored at 4°C in the dark until acquisition. Antibody capture beads (BD Biosciences) were used to prepare individual compensation controls for each antibody used in the experiment. ArC Amine Reactive beads (ThermoFisher Scientific) were used to generate a singly stained compensation control for the aqua amine-reactive viability dye.

For each stimulation condition, a minimum of 250,000 total events were acquired using a modified LSRII (BD Immunocytometry Systems). Data analysis was performed using FlowJo (TreeStar) software. Gating strategy is provided in the supplementary materials (S1 Fig). Reported antigen-specific data have been corrected for background based on the negative (no peptide) control, and only responses with a total frequency twice the negative control and above 0.01% of total memory CD8^+^ T cells (after background subtraction) were considered to be positive responses. By analyzing the data in this way, we examined cytolytic protein production resulting from antigen-specific stimulation and ensured that its expression was considered only within responding CD8^+^ T cells expressing at least one other functional parameter. Whereas IFN-γ, CD107a, and MIP-1α were used to identify antigen-specific CD8^+^ T cells for some donors, only IFN-γ and CD107a were used consistently for all donors and figures depicting antigen-specific data were derived from analysis of cells expressing these two markers unless otherwise noted.

### Statistical analysis

All statistical analysis was performed using Stata (version 14.0). Graphs were generated using Stata or GraphPad Prism (version 5.0a). Generalized estimating equations (GEEs) with robust variances were used to test for changes while adjusting for repeated measurements on the same individuals [75]. In instances where many values were at 100% a random-effects tobit regression model was used to do a combined analysis of the percent of data points at 100% versus differences in values for data points below 100%. *P* values were Holm-adjusted for multiple comparisons. Bars represent approximations of the means generated by the models. Lowess smoothers were used to represent the mean over time for longitudinal data. Correlations were determined using Spearman’s rank correlation test (non-parametric; two-tailed).

## Supporting Information

S1 Fig Gating strategy for the polychromatic flow cytometric staining panel. General gating strategy for a representative donor to identify total CD8^+^ T cells, CCR7 and CD45RO memory subsets, total perforin^+^ cells, T-bet^+^ cells, Eomes^+^ cells, and responding cells (IFN-γ^+^, CD107a^+^, or MIP-1α) following stimulation with Gag peptides. Gag-specific cells were assessed to be perforin^+^ if they expressed perforin in conjunction with IFN-γ, CD107a, or MIP-1α.
(PDF)

S2 Fig Correlation analyses of HIV plasma viral load versus total memory and perforin^+^ CD8^+^ T cells. (**A**) Correlation analyses for total memory CD8^+^ T cells with (from left to right) peak viral load plotted against acute memory frequency (*n* = 11), acute viral load plotted against acute memory frequency (*n* = 15), set point viral load plotted against set point memory frequency (*n* = 30), and chronic viral load plotted against chronic memory frequency (*n* = 18). (**B**) Correlation analyses for total perforin^+^ CD8^+^ T cells with (from left to right) peak viral load plotted against acute perforin^+^ frequency (*n* = 11), acute viral load plotted against acute perforin^+^ frequency (*n* = 15), set point viral load plotted against set point perforin^+^ frequency (*n* = 30), and chronic viral load plotted against chronic perforin^+^ frequency (*n* = 18). Spearman’s rank correlation test was used to determine significance.
(PDF)

S3 Fig Timing of HIV-seronegative study participant samples. Timing of samples for HIV-seronegative healthy donors relative to vaccination with live attenuated vaccinia virus (**A**), live attenuated yellow fever virus (**B**), or experimental infection with influenza (**C**).
(PDF)

S4 Fig Dynamics of CD8+ T cell memory subsets following infection with HIV, vaccinia virus, yellow fever virus, or influenza. Proportion of central memory CD8^+^ T cells for longitudinal time points from donors either naturally infected with HIV (*n* = 11; **A**), vaccinated with attenuated vaccinia virus (Dryvax, *n* = 8; **B**), vaccinated with live yellow fever virus (YFV-17D, *n* = 10; **C**), or experimentally infected with influenza (strain H1N1, *n* = 10; **D**). Proportion of effector memory CD8^+^ T cells following infection with HIV (**E**), vaccinia (**F**), yellow fever (**G**) or influenza (**H**). Proportion of effector CD8^+^ T cells following infection with HIV (**I**), vaccinia (**J**), yellow fever (**K**) or influenza (**L**). Pre = pre-infection time points. Pre-infection time points for HIV, vaccinia, and YFV, were set as day 0 for analysis. All data represent direct *ex vivo* assessment with no *in vitro* stimulation. Statistics based on a GEE population-averaged model with Holm adjusted *P* value. Bars represent approximations of the means generated by the models.
(PDF)

S5 Fig Activation of peripheral memory CD8^+^ T cells for HIV-negative individuals following vaccination with live attenuated vaccinia virus or live attenuated yellow fever virus. (**A**) Representative flow cytometric plots of perforin versus HLA-DR for two vaccinia-infected subjects. (**B**) Proportion of memory CD8^+^ T cells that express HLA-DR over time from infection for all vaccinia-vaccinated subjects (*n* = 8). (**C**) Representative flow cytometric plots of perforin versus HLA-DR for two yellow fever-vaccinated subjects. (**D**) Proportion of memory CD8^+^ T cells that express HLA-DR over time from infection for five yellow fever-infected subjects (*n* = 5). All data represent direct *ex vivo* assessment with no *in vitro* stimulation.
(PDF)

S6 Fig Magnitude and functionality of Nef-specific responses over time. (**A**) Frequency of Nef-specific CD8^+^ T cells within the memory CD8^+^ T cell pool over time as determined by measurement of IFN-γ expression or degranulation (CD107a) in response to peptide stimulation (*n* = 32). (**B**) Proportion of total responding Nef-specific CD8^+^ T cells that have upregulated IFN-γ or degranulated at acute (squares; *n* = 25), and chronic (triangles; *n* = 15) HIV time points. (**C**) Nef-specific CD8^+^ T cells (red) overlaid on total memory CD8^+^ T cells (black) for a representative donor. Percentages represent frequency of responding Nef-specific cells within a quadrant. § Day 420 sample was acquired and analyzed at a later date than earlier samples resulting in a different gating scheme. For consistency gates were set using naïve (CCR7^+^CD45RO^-^) CD8^+^ T cells. (**D**) Proportion of total responding Nef-specific CD8^+^ T cells that upregulated perforin in response to peptide stimulation (*n* = 25). Statistics based on a GEE population-averaged model with Holm adjusted *P* value or random-effects tobit regression. Bars represent approximations of the means generated by the models. Lowess smoothers were used to represent the mean over time for longitudinal data.
(PDF)

S7 Fig Magnitude and functionality of HIV-specific responses over time using IFN-γ, CD107a, and MIP-1α. Frequency of Gag-specific CD8^+^ T cells within the memory CD8^+^ T cell pool over time as determined by measurement of IFN-γ and CD107a (*n* = 28; **A**) or IFN-γ, CD107a, and MIP-1α (*n* = 22; **B**) in response to peptide stimulation. (**C**) Proportion of total responding Gag-specific CD8^+^ T cells that have upregulated IFN-γ, degranulated, or upregulated MIP-1α at acute (23–100 days; *n* = 18) and chronic (>365 days; n = 28) time points. Proportion of total responding Gag-specific CD8^+^ T cells that upregulated perforin in response to peptide stimulation using IFN-γ and CD107a (*n* = 28; **D**) or IFN-γ, CD107a, and MIP-1α (*n* = 17; **E**) to identify responding cells. Statistics based on a GEE population-averaged model with Holm adjusted *P* value or random-effects tobit regression. Bars represent approximations of the means generated by the models. Lowess smoothers were used to represent the mean over time for longitudinal data.
(PDF)

S8 Fig Differential T-bet and Eomes expression levels within total perforin^+^ CD8^+^ T cells for healthy donors and following HIV infection. (**A**) Representative flow cytometric plots of T-bet and Eomes expression for perforin^+^ CD8^+^ T cells from the pre- (day -159), acute (day 28), and chronic (day 434) infection time points for one donor. (**B**) T-bet and Eomes expression by perforin^+^ CD8^+^ T cells for all healthy donors (circles; *n* = 29), acute HIV time points (squares; *n* = 21), and chronic HIV time points (triangles; *n* = 23). All data represent direct *ex vivo* assessment with no *in vitro* stimulation. ** denotes a *P* value < 0.01 and *** denotes a *P* value < 0.001. Statistics based on a GEE population-averaged model with Holm adjusted *P* value. Bars represent approximations of the means generated by the models.
(PDF)

S9 Fig Correlation analyses of viral load versus total memory CD8^+^ T cell T-bet and Eomes subsets. (**A-D**) Correlation analyses for memory CD8^+^ T cell T-bet and Eomes subsets with (from left to right) peak viral load plotted against acute subset frequency (*n* = 11), set point viral load plotted against acute subset frequency (*n* = 15 for T-bet^+^ and 11 for other subsets), set point viral load plotted against set point subset frequency (*n* = 30 for T-bet^+^ and 23 for other subsets), and chronic viral load plotted against chronic subset frequency (*n* = 18) for T-bet^+^ (**A**), Eomes^+^ (**B**), T-bet^+^Eomes^+^ (**C**), and T-bet^-^Eomes^-^ cells (**D**). Spearman’s rank correlation test was used to determine significance.
(PDF)

S10 Fig Correlation analyses of viral load versus perforin^+^ CD8^+^ T cell T-bet and Eomes subsets. (**A-D**) Correlation analyses for perforin^+^ CD8^+^ T cell T-bet and Eomes subsets with (from left to right) peak viral load plotted against acute subset frequency (*n* = 11), set point viral load plotted against acute subset frequency (*n* = 15 for T-bet^+^ and 11 for other subsets), set point viral load plotted against set point subset frequency (*n* = 30 for T-bet^+^ and 23 for other subsets), and chronic viral load plotted against chronic subset frequency (*n* = 18) for T-bet^+^ (**A**), Eomes^+^ (**B**), T-bet^+^Eomes^+^ (**C**), and T-bet^-^Eomes^-^ cells (**D**). Spearman’s rank correlation test was used to determine significance.
(PDF)

S11 Fig Loss of T-bet and Eomes expression by perforin^+^ or HLA-DR^+^ cells over the course of infection with yellow fever, vaccinia, or HIV. (**A**) T-bet and Eomes expression over the course of yellow fever vaccination for perforin^+^ (top) or HLA-DR^+^ (bottom) memory CD8^+^ T cells. Representative flow cytometric plots for one donor; T-bet^+^Eomes^+^ and T-bet^-^Eomes^-^ subsets shown for all five donors. (**B**) T-bet and Eomes expression over the course of vaccinia vaccination for perforin^+^ (top) or HLA-DR^+^ (bottom) memory CD8^+^ T cells. Representative flow cytometric plots for one donor; T-bet^+^Eomes^+^ and T-bet^-^Eomes^-^ subsets shown for all eight donors. (**C**) T-bet^+^Eomes^+^ and T-bet^-^Eomes^-^ subsets for perforin^+^ (top) or HLA-DR^+^ (bottom) memory CD8^+^ T cells from four RV217 donors. All data represent direct *ex vivo* assessment with no *in vitro* stimulation.
(PDF)

S12 Fig Correlation analyses of viral load versus Gag-specific CD8^+^ T cell magnitude and perforin expression. (**A**) Correlation analyses for Gag-specific CD8^+^ T cells with (from left to right) peak viral load plotted against acute frequency (*n* = 11), acute viral load plotted against acute frequency (*n* = 15), set point viral load plotted against set point frequency (*n* = 30), and chronic viral load plotted against chronic frequency (*n* = 19). (**B**) Correlation analyses for the proportion of Gag-specific CD8^+^ T cells that are perforin^+^ with (from left to right) peak viral load plotted against acute proportion (*n* = 7), acute viral load plotted against acute proportion (*n* = 10), set point viral load plotted against set point proportion (*n* = 25), and chronic viral load plotted against chronic proportion (*n* = 16). Only those subjects for whom a Gag-specific response was detected are plotted. Spearman’s rank correlation test was used to determine significance.
(PDF)

S13 Fig Correlation analyses of viral load versus Gag-specific CD8^+^ T cell T-bet and Eomes subsets. (**A-E**) Correlation analyses for Gag-specific CD8^+^ T cell T-bet and Eomes subsets with (from left to right) peak viral load plotted against acute subset frequency (*n* = 7), acute viral load plotted against acute subset frequency (*n* = 10 for T-bet^+^ and T-bet^Hi^ subsets and 7 for all other subsets), set point viral load plotted against set point subset frequency (*n* = 27 for T-bet^+^ and T-bet^Hi^ subsets and 18 for all other subsets), and chronic viral load plotted against chronic subset frequency (*n* = 17) for T-bet^+^ (**A**), T-bet^Hi^ (**B**), Eomes^+^ (**C**), T-bet^Hi^Eomes^+^ (**D**), and T-bet^Lo^Eomes^-^ cells (**E**). Spearman’s rank correlation test was used to determine significance.
(PDF)

S1 Table Acute/early HIV cohort demographics. (PDF)
