# Supplementary figures and images for: Temporal Dynamics of CD8+ T Cell Effector Responses during Primary HIV Infection
Source: PLoS Pathog. 2016 Aug 3;12(8):e1005805. doi: 10.1371/journal.ppat.1005805 (PMC4972399; doi:10.1371/journal.ppat.1005805)

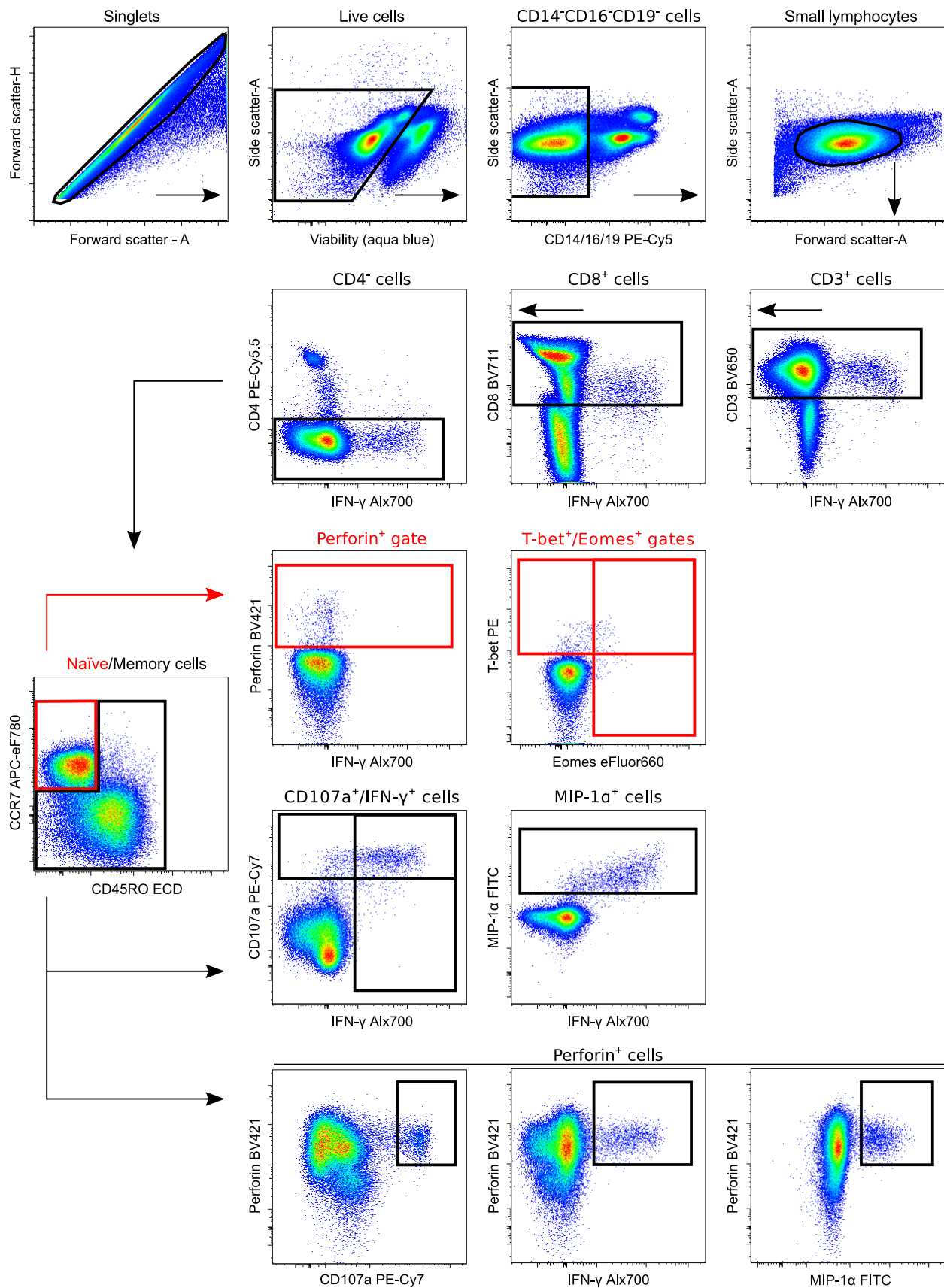

Supplement: S1 Fig — General gating strategy for a representative donor to identify total CD8+ T cells, CCR7 and CD45RO memory subsets, total perforin+ cells, T-bet+ cells, Eomes+ cells, and responding cells (IFN-γ+, CD107a+, or MIP-1α) following stimulation with Gag peptides. Gag-specific cells were assessed to be perforin+ if they expressed perforin in conjunction with IFN-γ, CD107a, or MIP-1α. (PDF) [file ppat.1005805.s001.pdf]

**A**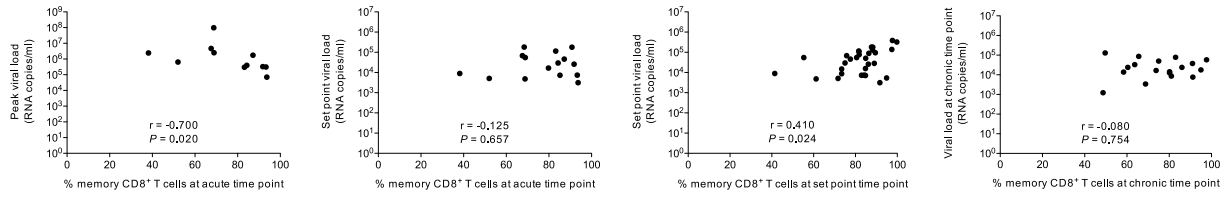**B**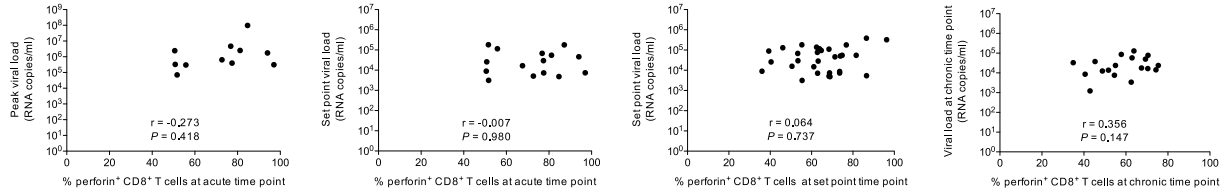

Supplement: S2 Fig — (A) Correlation analyses for total memory CD8+ T cells with (from left to right) peak viral load plotted against acute memory frequency (n = 11), acute viral load plotted against acute memory frequency (n = 15), set point viral load plotted against set point memory frequency (n = 30), and chronic viral load plotted against chronic memory frequency (n = 18). (B) Correlation analyses for total perforin+ CD8+ T cells with (from left to right) peak viral load plotted against acute perforin+ frequency (n = 11), acute viral load plotted against acute perforin+ frequency (n = 15), set point viral load plotted against set point perforin+ frequency (n = 30), and chronic viral load plotted against chronic perforin+ frequency (n = 18). Spearman’s rank correlation test was used to determine significance. (PDF) [file ppat.1005805.s002.pdf]

**A**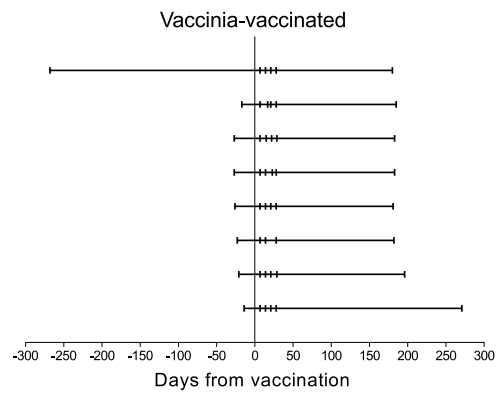**B**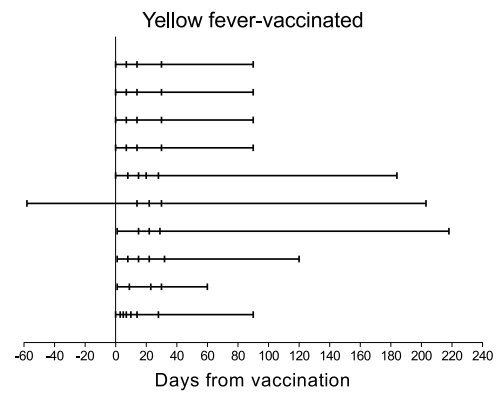**C**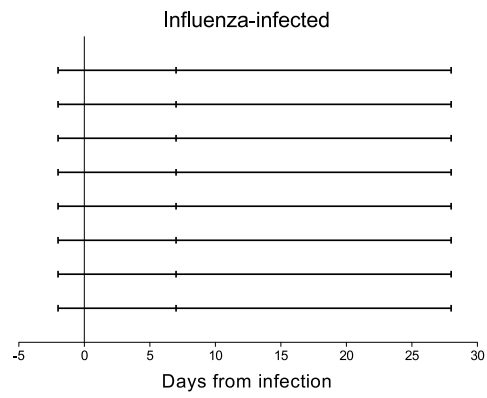

Supplement: S3 Fig — Timing of samples for HIV-seronegative healthy donors relative to vaccination with live attenuated vaccinia virus (A), live attenuated yellow fever virus (B), or experimental infection with influenza (C). (PDF) [file ppat.1005805.s003.pdf]

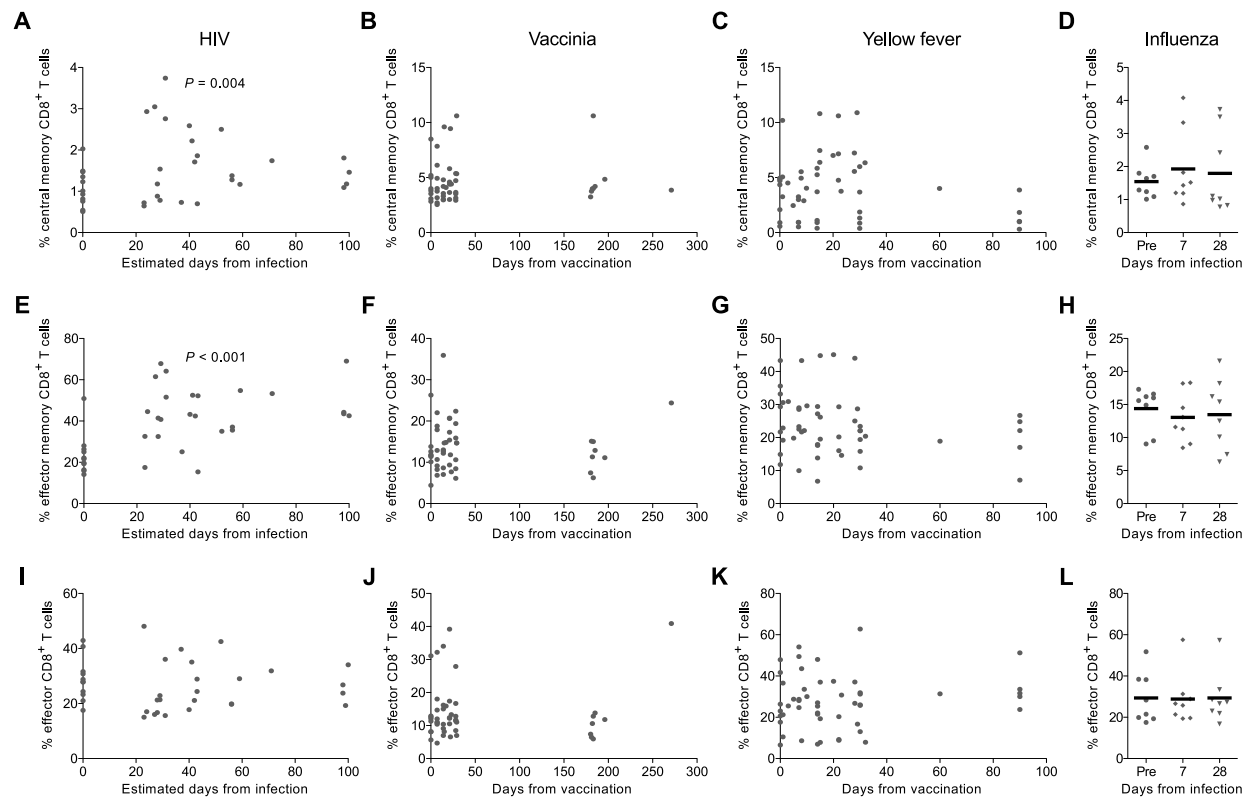

Supplement: S4 Fig — Proportion of central memory CD8+ T cells for longitudinal time points from donors either naturally infected with HIV (n = 11; A), vaccinated with attenuated vaccinia virus (Dryvax, n = 8; B), vaccinated with live yellow fever virus (YFV-17D, n = 10; C), or experimentally infected with influenza (strain H1N1, n = 10; D). Proportion of effector memory CD8+ T cells following infection with HIV (E), vaccinia (F), yellow fever (G) or influenza (H). Proportion of effector CD8+ T cells following infection with HIV (I), vaccinia (J), yellow fever (K) or influenza (L). Pre = pre-infection time points. Pre-infection time points for HIV, vaccinia, and YFV, were set as day 0 for analysis. All data represent direct ex vivo assessment with no in vitro stimulation. Statistics based on a GEE population-averaged model with Holm adjusted P value. Bars represent approximations of the means generated by the models. (PDF) [file ppat.1005805.s004.pdf]

### Vaccinia virus vaccination

**A**

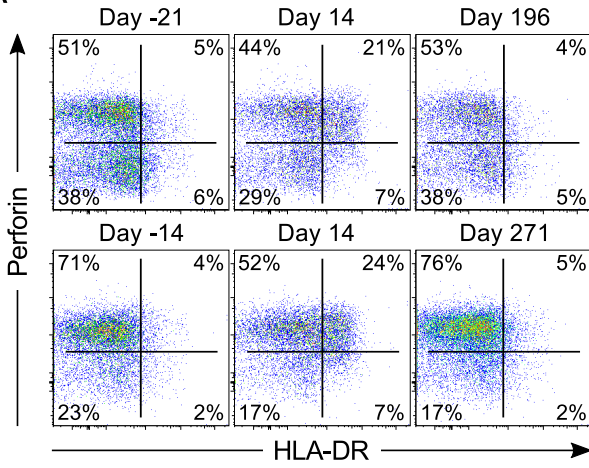

**B**

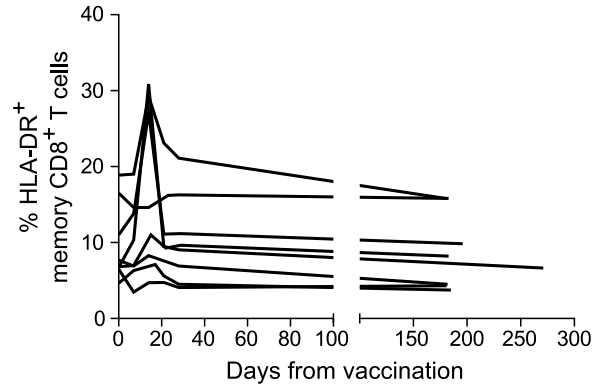

### Yellow fever virus vaccination

**C**

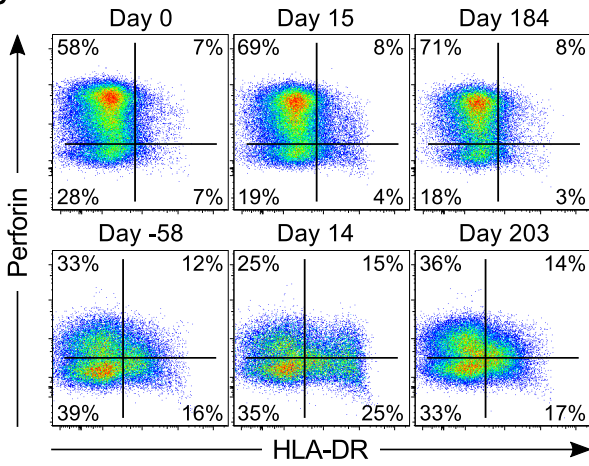

**D**

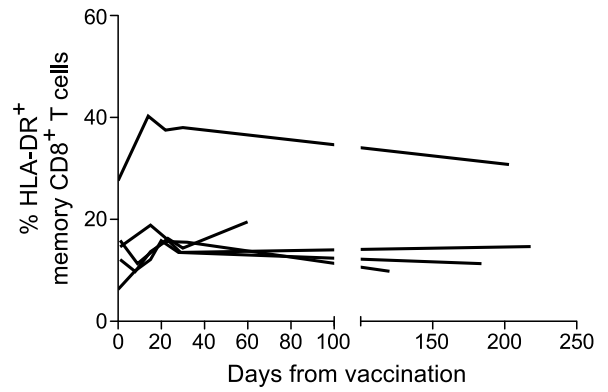

Supplement: S5 Fig — (A) Representative flow cytometric plots of perforin versus HLA-DR for two vaccinia-infected subjects. (B) Proportion of memory CD8+ T cells that express HLA-DR over time from infection for all vaccinia-vaccinated subjects (n = 8). (C) Representative flow cytometric plots of perforin versus HLA-DR for two yellow fever-vaccinated subjects. (D) Proportion of memory CD8+ T cells that express HLA-DR over time from infection for five yellow fever-infected subjects (n = 5). All data represent direct ex vivo assessment with no in vitro stimulation. (PDF) [file ppat.1005805.s005.pdf]

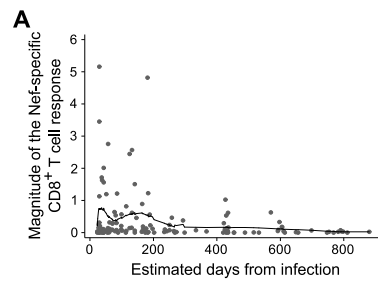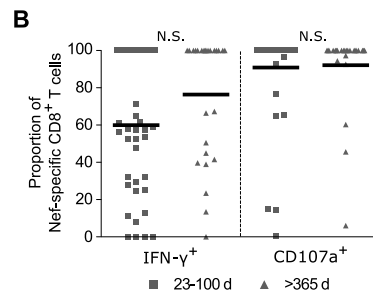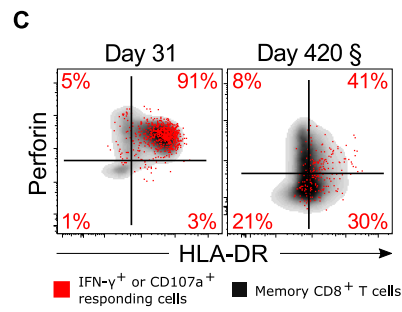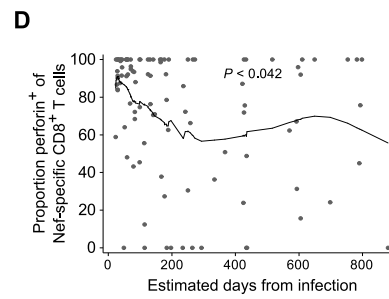

Supplement: S6 Fig — (A) Frequency of Nef-specific CD8+ T cells within the memory CD8+ T cell pool over time as determined by measurement of IFN-γ expression or degranulation (CD107a) in response to peptide stimulation (n = 32). (B) Proportion of total responding Nef-specific CD8+ T cells that have upregulated IFN-γ or degranulated at acute (squares; n = 25), and chronic (triangles; n = 15) HIV time points. (C) Nef-specific CD8+ T cells (red) overlaid on total memory CD8+ T cells (black) for a representative donor. Percentages represent frequency of responding Nef-specific cells within a quadrant. § Day 420 sample was acquired and analyzed at a later date than earlier samples resulting in a different gating scheme. For consistency gates were set using naïve (CCR7+CD45RO-) CD8+ T cells. (D) Proportion of total responding Nef-specific CD8+ T cells that upregulated perforin in response to peptide stimulation (n = 25). Statistics based on a GEE population-averaged model with Holm adjusted P value or random-effects tobit regression. Bars represent approximations of the means generated by the models. Lowess smoothers were used to represent the mean over time for longitudinal data. (PDF) [file ppat.1005805.s006.pdf]

**A**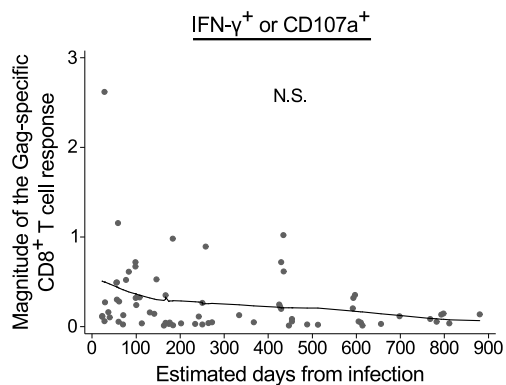**B**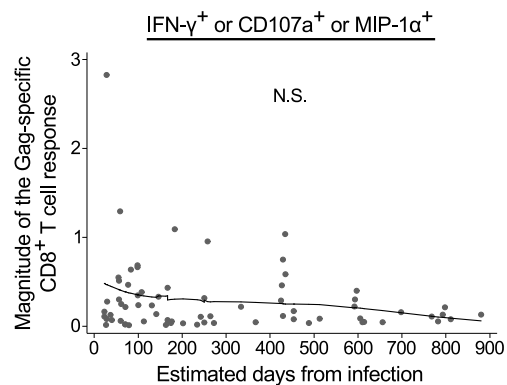**C**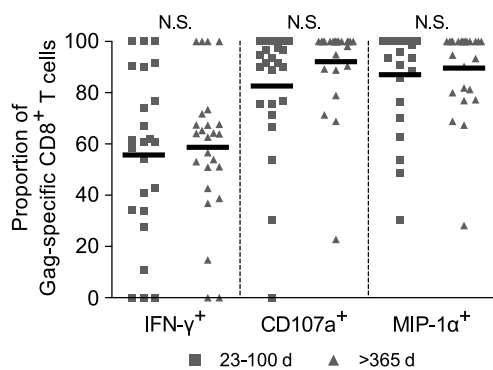**D**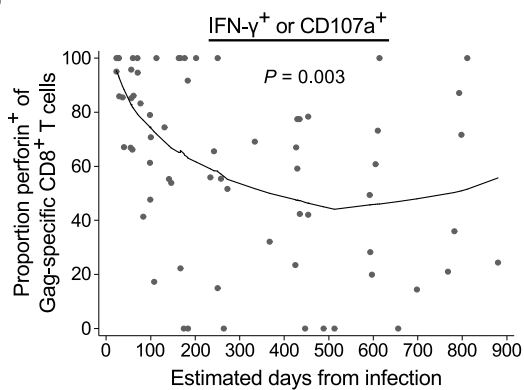**E**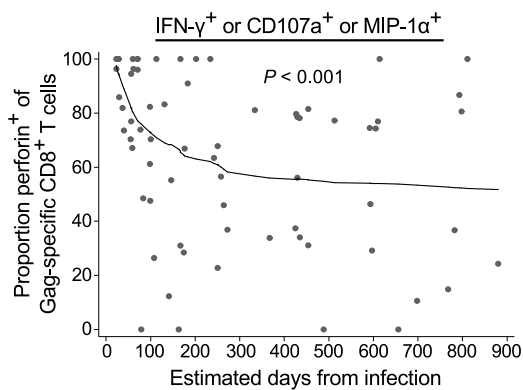

Supplement: S7 Fig — Frequency of Gag-specific CD8+ T cells within the memory CD8+ T cell pool over time as determined by measurement of IFN-γ and CD107a (n = 28; A) or IFN-γ, CD107a, and MIP-1α (n = 22; B) in response to peptide stimulation. (C) Proportion of total responding Gag-specific CD8+ T cells that have upregulated IFN-γ, degranulated, or upregulated MIP-1α at acute (23–100 days; n = 18) and chronic (>365 days; n = 28) time points. Proportion of total responding Gag-specific CD8+ T cells that upregulated perforin in response to peptide stimulation using IFN-γ and CD107a (n = 28; D) or IFN-γ, CD107a, and MIP-1α (n = 17; E) to identify responding cells. Statistics based on a GEE population-averaged model with Holm adjusted P value or random-effects tobit regression. Bars represent approximations of the means generated by the models. Lowess smoothers were used to represent the mean over time for longitudinal data. (PDF) [file ppat.1005805.s007.pdf]

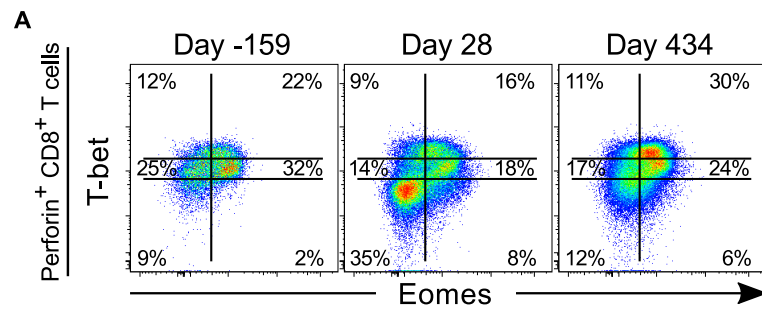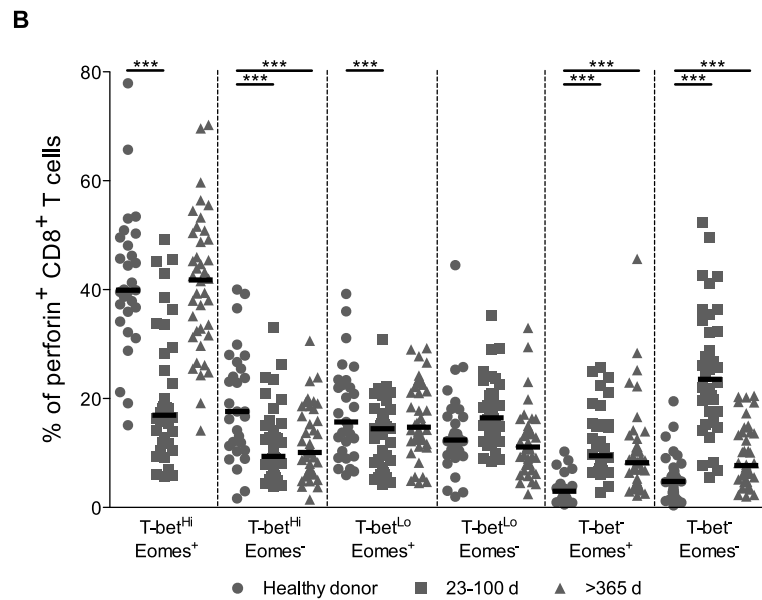

Supplement: S8 Fig — (A) Representative flow cytometric plots of T-bet and Eomes expression for perforin+ CD8+ T cells from the pre- (day -159), acute (day 28), and chronic (day 434) infection time points for one donor. (B) T-bet and Eomes expression by perforin+ CD8+ T cells for all healthy donors (circles; n = 29), acute HIV time points (squares; n = 21), and chronic HIV time points (triangles; n = 23). All data represent direct ex vivo assessment with no in vitro stimulation. ** denotes a P value < 0.01 and *** denotes a P value < 0.001. Statistics based on a GEE population-averaged model with Holm adjusted P value. Bars represent approximations of the means generated by the models. (PDF) [file ppat.1005805.s008.pdf]

**A**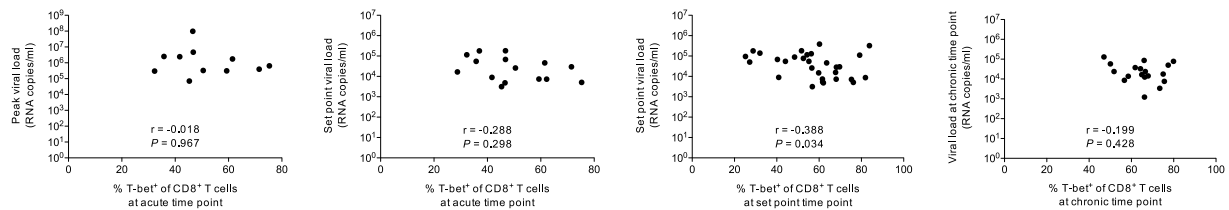**B**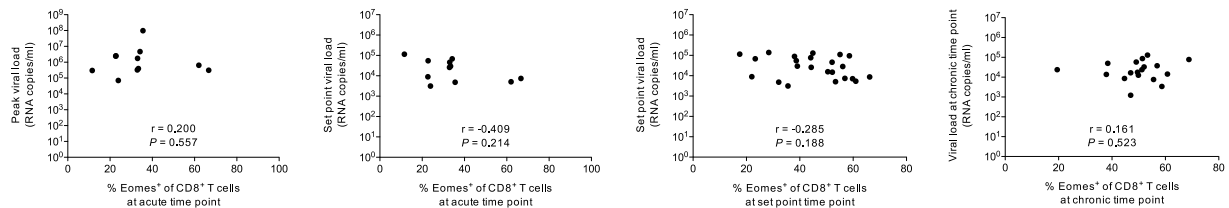**C**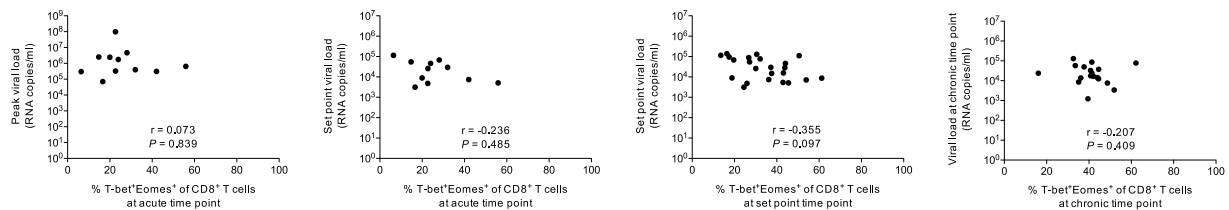**D**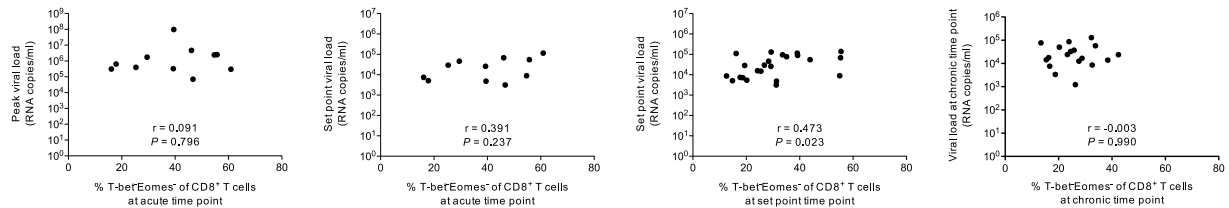

Supplement: S9 Fig — (A-D) Correlation analyses for memory CD8+ T cell T-bet and Eomes subsets with (from left to right) peak viral load plotted against acute subset frequency (n = 11), set point viral load plotted against acute subset frequency (n = 15 for T-bet+ and 11 for other subsets), set point viral load plotted against set point subset frequency (n = 30 for T-bet+ and 23 for other subsets), and chronic viral load plotted against chronic subset frequency (n = 18) for T-bet+ (A), Eomes+ (B), T-bet+Eomes+ (C), and T-bet-Eomes- cells (D). Spearman’s rank correlation test was used to determine significance. (PDF) [file ppat.1005805.s009.pdf]

**A**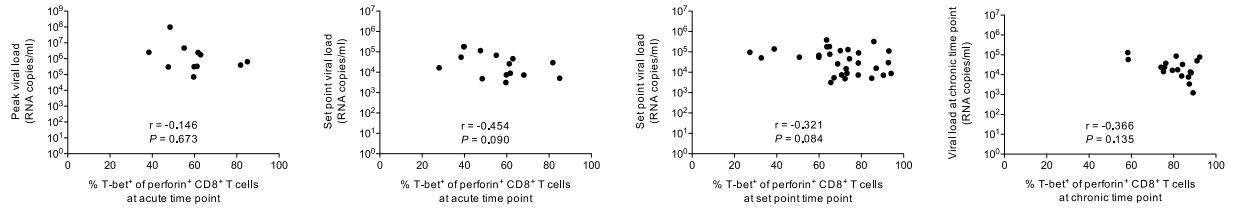**B**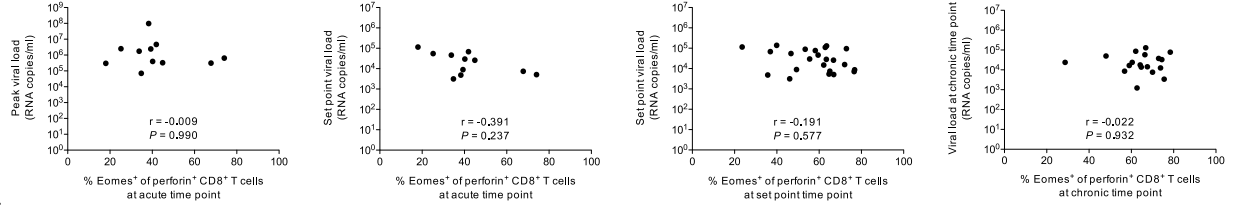**C**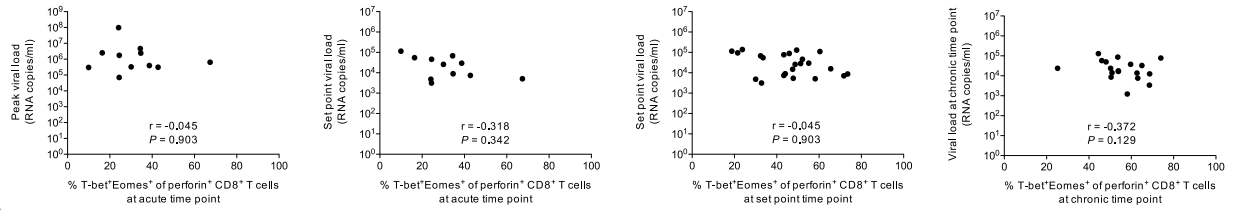**D**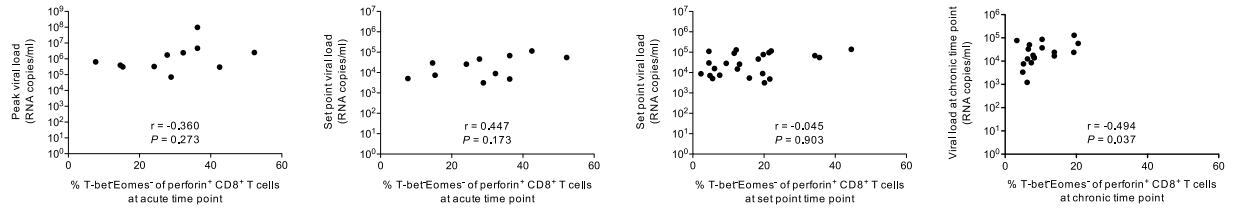

Supplement: S10 Fig — (A-D) Correlation analyses for perforin+ CD8+ T cell T-bet and Eomes subsets with (from left to right) peak viral load plotted against acute subset frequency (n = 11), set point viral load plotted against acute subset frequency (n = 15 for T-bet+ and 11 for other subsets), set point viral load plotted against set point subset frequency (n = 30 for T-bet+ and 23 for other subsets), and chronic viral load plotted against chronic subset frequency (n = 18) for T-bet+ (A), Eomes+ (B), T-bet+Eomes+ (C), and T-bet-Eomes- cells (D). Spearman’s rank correlation test was used to determine significance. (PDF) [file ppat.1005805.s010.pdf]

## Yellow fever virus vaccination

**A**

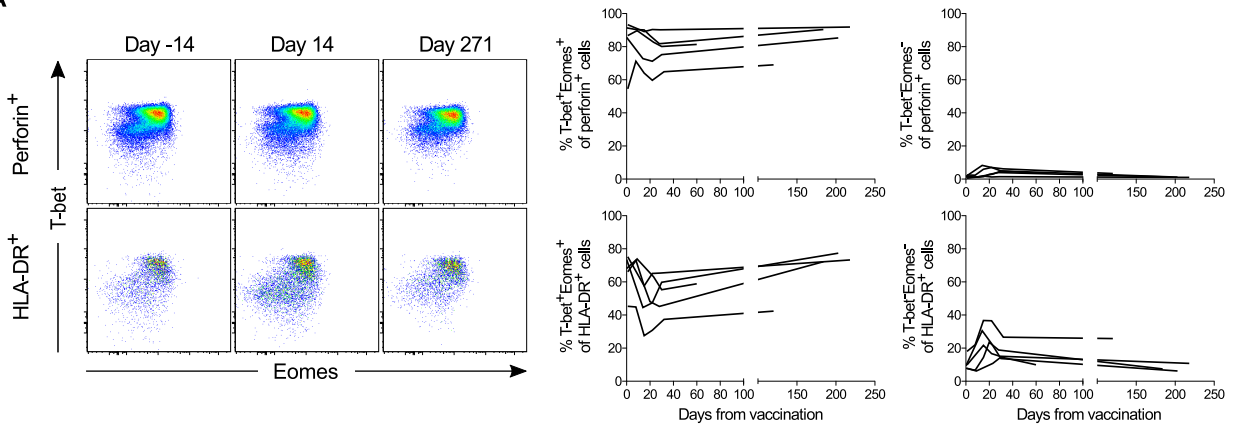

## Vaccinia virus vaccination

**B**

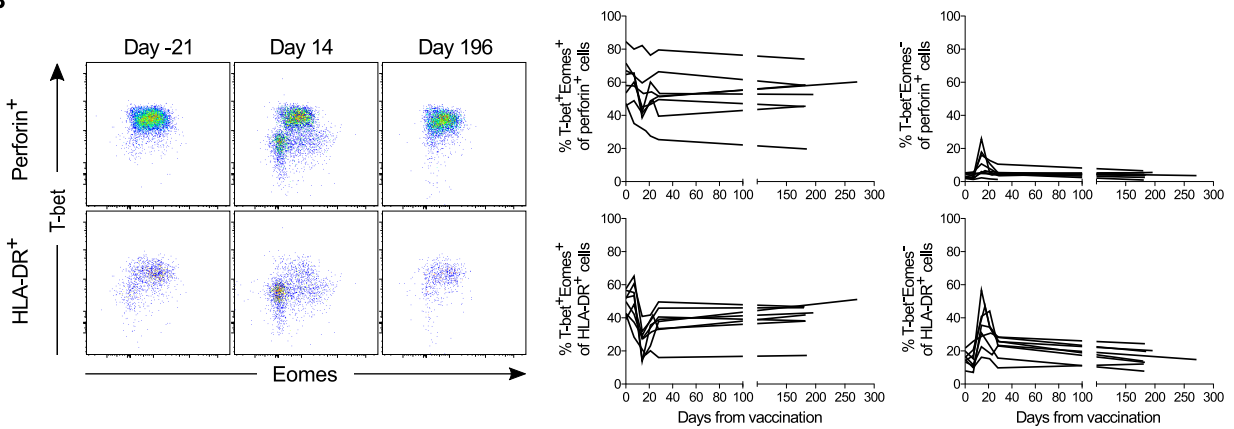

## HIV infection

**C**

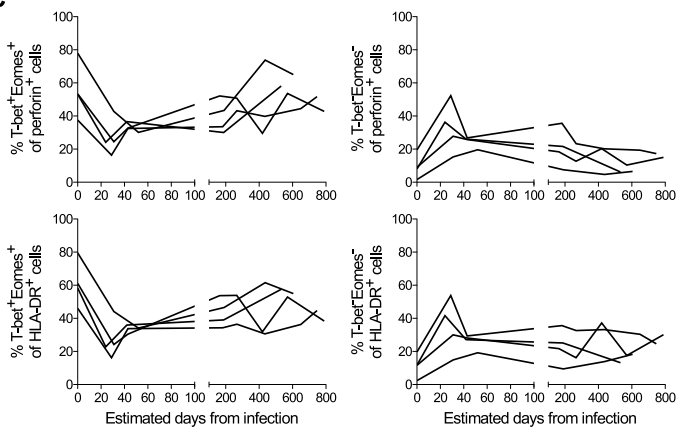

Supplement: S11 Fig — (A) T-bet and Eomes expression over the course of yellow fever vaccination for perforin+ (top) or HLA-DR+ (bottom) memory CD8+ T cells. Representative flow cytometric plots for one donor; T-bet+Eomes+ and T-bet-Eomes- subsets shown for all five donors. (B) T-bet and Eomes expression over the course of vaccinia vaccination for perforin+ (top) or HLA-DR+ (bottom) memory CD8+ T cells. Representative flow cytometric plots for one donor; T-bet+Eomes+ and T-bet-Eomes- subsets shown for all eight donors. (C) T-bet+Eomes+ and T-bet-Eomes- subsets for perforin+ (top) or HLA-DR+ (bottom) memory CD8+ T cells from four RV217 donors. All data represent direct ex vivo assessment with no in vitro stimulation. (PDF) [file ppat.1005805.s011.pdf]

**A**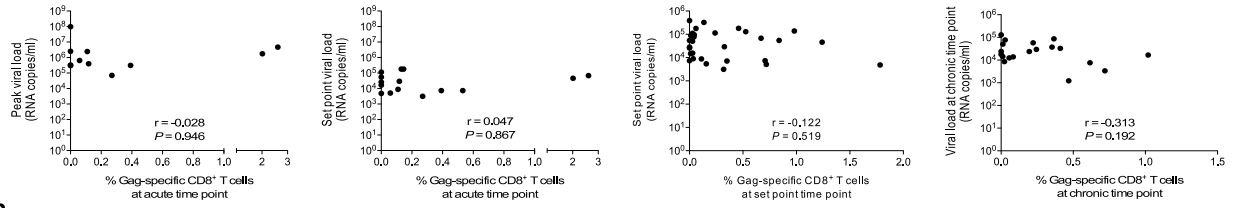**B**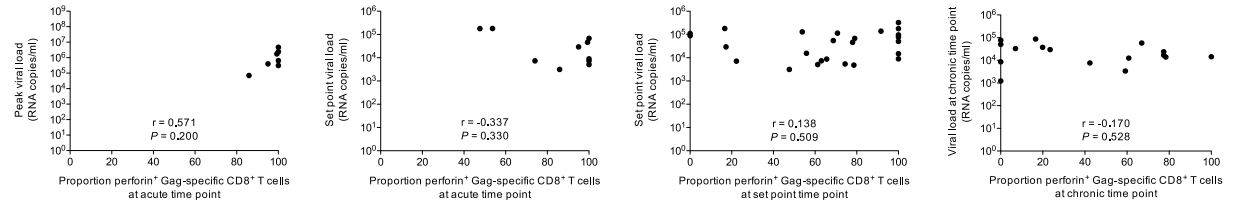

Supplement: S12 Fig — (A) Correlation analyses for Gag-specific CD8+ T cells with (from left to right) peak viral load plotted against acute frequency (n = 11), acute viral load plotted against acute frequency (n = 15), set point viral load plotted against set point frequency (n = 30), and chronic viral load plotted against chronic frequency (n = 19). (B) Correlation analyses for the proportion of Gag-specific CD8+ T cells that are perforin+ with (from left to right) peak viral load plotted against acute proportion (n = 7), acute viral load plotted against acute proportion (n = 10), set point viral load plotted against set point proportion (n = 25), and chronic viral load plotted against chronic proportion (n = 16). Only those subjects for whom a Gag-specific response was detected are plotted. Spearman’s rank correlation test was used to determine significance. (PDF) [file ppat.1005805.s012.pdf]

**A**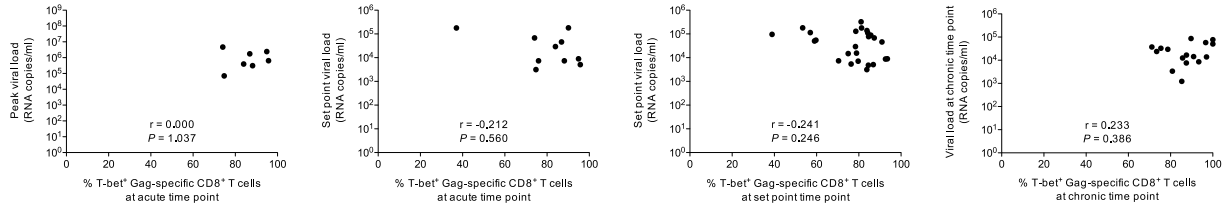**B**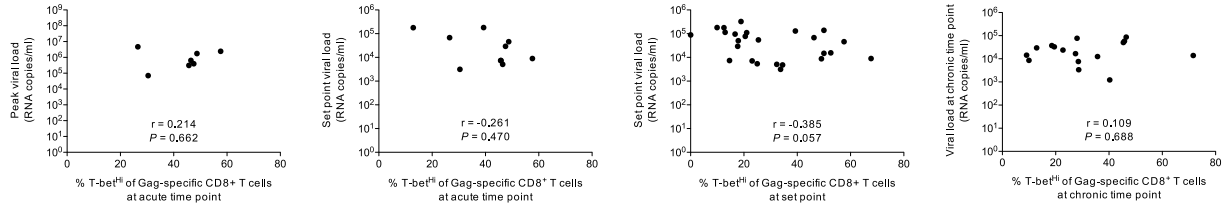**C**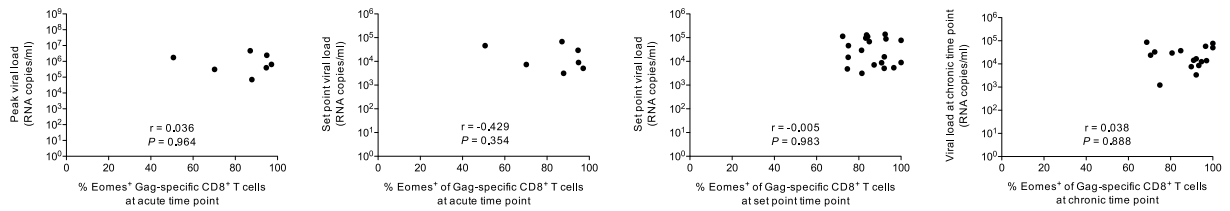**D**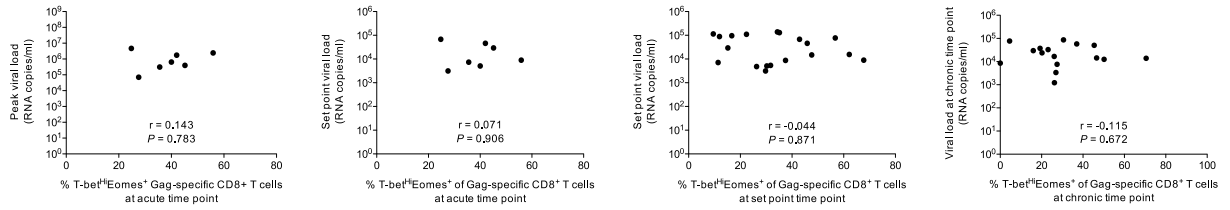**E**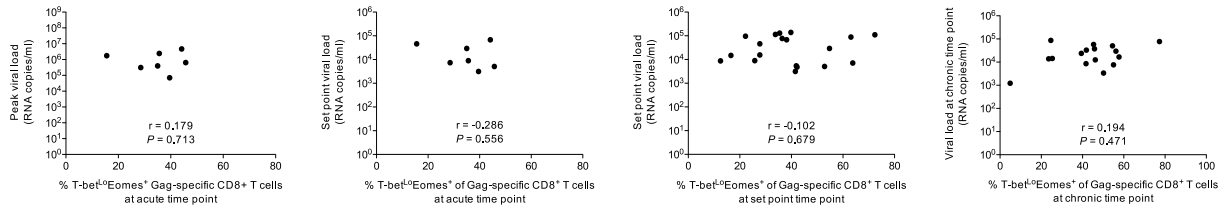

Supplement: S13 Fig — (A-E) Correlation analyses for Gag-specific CD8+ T cell T-bet and Eomes subsets with (from left to right) peak viral load plotted against acute subset frequency (n = 7), acute viral load plotted against acute subset frequency (n = 10 for T-bet+ and T-betHi subsets and 7 for all other subsets), set point viral load plotted against set point subset frequency (n = 27 for T-bet+ and T-betHi subsets and 18 for all other subsets), and chronic viral load plotted against chronic subset frequency (n = 17) for T-bet+ (A), T-betHi (B), Eomes+ (C), T-betHiEomes+ (D), and T-betLoEomes- cells (E). Spearman’s rank correlation test was used to determine significance. (PDF) [file ppat.1005805.s013.pdf]
