# Supplementary material for: Temporal Dynamics of CD8+ T Cell Effector Responses during Primary HIV Infection
Source: PLoS Pathog. 2016 Aug 3;12(8):e1005805. doi: 10.1371/journal.ppat.1005805 (PMC4972399; doi:10.1371/journal.ppat.1005805)
Supplement: S1 Table — (PDF) [file ppat.1005805.s014.pdf]

Study[a]	Donor ID	Gender	Age	Country	Days Pre-infection[b]	Days Infected[b,c]	RNA copies/ml[c]	Clade
CHAVI	700010621	M	40	USA	-	125	387344	N.D.
CHAVI	700010717	F	30	USA	-	29	750000	N.D.
CHAVI	701010248	M	24	USA	-	29	2485	N.D.
CHAVI	702010157	M	21	Malawi	-	29	750000	N.D.
CHAVI	702010176	M	21	Malawi	-	67	750000	N.D.
CHAVI	702010202	M	30	Malawi	-	293	64114	N.D.
CHAVI	702010280	M	21	Malawi	-	125	295688	N.D.
CHAVI	703010200	M	24	Malawi	-	38	125291	C
CHAVI	703010217	F	34	Malawi	-	29	102602	C
Montreal	ACT90233	M	31	Canada	-	68	11886	B
Montreal	ACT92900	M	31	Canada	-	61	8392	B
Montreal	GOL033G	M	29	Canada	-	77	94436	B
Montreal	GOL036G	M	59	Canada	-	71	181711	B
Montreal	GOL037G	M	41	Canada	-	60	60799	B
Montreal	GOL038G	M	44	Canada	-	55	4889	B
Montreal	HNDDRPI050	M	35	Canada	-	177	11785	B
Montreal	HNDDRPI060	M	32	Canada	-	174	87768	B
Montreal	HNDDRPI062	M	34	Canada	-	79	207458	B
Montreal	HNDDRPI063	M	55	Canada	-	52	108207	B
Montreal	HNDDRPI064	M	27	Canada	-	70	87887	B
Montreal	HNDDRPI070	M	33	Canada	-	61	145228	B
RV217	10220	F	33	Uganda	-197	31	309030	A1
RV217	10374	F	26	Uganda	-174	41	10965	A1D
RV217	20225	F	24	Kenya	-125	24	1122018	A1C
RV217	30507	F	34	Tanzania	-277	29	1122	B/CRF01
RV217	40067	M	27	Thailand	-441	27	229087	A1C
RV217	40094	M	19	Thailand	-237	29	2511886	CRF01_AE
RV217	40123	M	23	Thailand	-238	31	630957	CRF01_AE
RV217	40134	M	18	Thailand	-48	23	2454708	C/CRF01
RV217	40250	M	35	Thailand	-159	28	4677351	CRF01_AE
RV217	40283	M	23	Thailand	-456	23	398107	B
RV217	40353	M	21	Thailand	-27	28	162181	B

[a] CHAVI = Center for HIV/AIDS Vaccine Immunology, Montreal = McGill University Health Center, RV217 = US Military HIV Research Program.

[b] Estimated days from infection was based on Fiebig staging at first positive visit (CHAVI and RV217) or available serological parameters in combination with donor proposed date (Montreal).

[c] At first time point analyzed.
